# Research, Reading, and Publication Habits of Nurses and Nursing Students Applied to Impact Journals: International Multicentre Study

**DOI:** 10.3390/ijerph20064697

**Published:** 2023-03-07

**Authors:** María Begoña Sánchez-Gómez, Gonzalo Duarte-Clíments, Juan Gómez-Salgado, María Elena González-Pacheco, María Elisa de Castro-Peraza, María Mercedes Novo-Muñoz, José Ángel Rodríguez-Gómez, José Ramón Martínez-Riera, Rafaella Pessoa-Moreira, Maria do Rosario Martins, Paloma Echevarría-Pérez, Ana Isabel Bonilla-Calero

**Affiliations:** 1 Cátedra de Enfermería, Universidad de la Laguna, 38200 San Cristobal de La Laguna, Spain; 2 Cieza Este Health Centre, Servicio Murciano de Salud, 30530 Murcia, Spain; 3 Department of Sociology, Social Work and Public Health, Faculty of Labour Sciences, University of Huelva, 21004 Huelva, Spain; 4 Safety and Health Postgraduate Program, Universidad Espíritu Santo, Guayaquil 092301, Ecuador; 5 Centro Universitario de la Ciénega, University of Guadalajara, Guadalajara 44100, Jalisco, Mexico; 6 Department of Nursing, University of La Laguna, 38200 Santa Cruz de Tenerife, Spain; 7 Department of Community Nursing, Preventive Medicine, Public Health and History of Science, University of Alicante, 03690 Alicante, Spain; 8 ICIS Instituto de Ciências da Saúde, Universidade da Integração Internacional da Lusofonia Afro-Brasileira (UNILAB), Sede Redenção/Ceará, Redencao 62790-000, CE, Brazil; 9 UICISA:E, Escola Superior de Saúde, Instituto Politécnico de Viana do Castelo, Rua Dom Moisés Alves Pinho 190, 4900-314 Viana do Castelo, Portugal; 10 Managing Board, Catholic University of Murcia, 30107 Murcia, Spain

**Keywords:** competence, impact factor, nursing, health sciences, journals

## Abstract

Publishing in JCR and SJR journals has become crucial for curricular development. Results from nursing investigations “compete” for publication in journals which are not specific to the field of care, affecting the academic development of these investigators. This phenomenon may lead to an ongoing adverse effect on nursing researchers and academics engaged in research in nursing care. The aim of this study was to evaluate habits regarding scientific literature consulting, the transfer of published material, and the citation of nursing investigations. A cross-sectional descriptive study by means of questionnaires was carried out, focusing on both Spanish and Portuguese nurses. The findings of the study reveal the following reasons for reading the scientific literature: that the language was understood; for learning and applying what was learnt; that the journal was of open access; for elaborating protocols and work procedures; and that the journal was indexed in scientific databases and in nursing databases. The reasons for reading, using, and publishing in journals were related to knowledge of the language and the associated usefulness of learning and applying knowledge. Creating a specific index of research publications in nursing will have a positive effect on the scientific production of caring methodologies.

## 1. Introduction

In recent years, the number of scientific journals has increased worldwide. De Solla Price [1] found that the growth of scientific information is occurring at a much faster pace than other social phenomena. Thus, every 10–15 years, the amount of existing information doubles with exponential growth.

To achieve dissemination, the journal needs to be indexed in a database. The publications indexed in both Clarivate Analytics’ Web of Science (WoS) and Elsevier’s Scopus are considered a reference for indexing. In these databases, journals are grouped according to their repercussion, impact, and prestige [2]. These factors are measured by the number of citations that a specific publication receives in a given period of time.

From the analysis of received citations, numerical indicators of quality are established via the so-called Impact Factor (IF). In the case of WoS, the Journal Citation Report [3], popularly known as JCR index, is generated as an indicator, and in the case of Scopus [4], Scimago Journal & Country Rank, the SJR index is generated. These indexes are tools designed to measure the quality of the works with respect to the number of citations that each scientific contribution obtains, based on the premise that the journals that receive the most citations are those of the highest quality.

The impact factor of an article is important for both the journal and the authors. For example, among the evaluation criteria for the accreditation of university professors in different quality assessment agencies, in most cases, the “Research experience” criterion is the one that obtains the most points in the accreditation process [5]. In this regard, the applicant’s scientific publications are evaluated, with special, and sometimes exclusive, interest in those indexed in the Web of Science (WoS) and Scopus databases.

These indicators related to the impact factor are also decisive for access to research grants and pre- and post-doctoral training [5]. Thus, in 2019, the Carlos III Health Institute [6] approved the call for proposals for grants for the strategic health action for 2017–2020, and the JCR was the key indicator in the evaluation of researchers.

The usefulness of assessing publications from the point of view of the impact factor has been generally assumed. However, many limitations are observed in its application [7,8], such as the lack of comparability between different disciplines, which could generate a certain tendency to self-citation or to ascribing the same value to any citation received. In the case of nursing, given that care is sometimes considered within the clinical sciences and sometimes within the social sciences, there is no specific indexing section in either WoS or Scopus [3,4,5,6,7,8]. This results in nursing publications competing for publication in journals that are not specific to the field of care.

Nonetheless, there are databases such as CINAHL [9] or CUIDEN [10] that are specific to the nursing discipline. CINAHL indexes more than six million nursing records and approximately 5500 journals [9] and provides real-time information on article metrics. CUIDEN indexes approximately 11,256 documents and 451 journals exclusively in the Ibero-American area [10], Spain, and Portugal. This database provides information on impact or repercussion indicators, activity, and information consumption.

All of the above issues are particularly burdensome for nurses. Nurse researchers are faced with the dilemma of deciding whether to publish in journals that generate impact for their careers or in journals that specialise in care and which are read by the majority of nurses. This may lead to a gap between what is researched, what is published, what is read, and what is transferred into practice in the field of care. In other words, it is possible that care research is under-published in the JCR and SJR indexes due to a phenomenon of adverse selection in this field of research. In addition, publications indexed in CINAHL or CUIDEN are not a priority for measuring impact.

This phenomenon may lead to an ongoing adverse effect on nursing researchers and academics engaged in research in nursing care. These professionals cannot compete with the current criteria for access to research funding or access to university places. If they do not publish in JCR and SJR journals, they do not receive accreditation, and without accreditation, there is no access to research institutions [11,12]. This means that research on care is discouraged and rendered invisible.

As a result, moving away from healthcare research has two additional consequences. First, without research, there is no improvement in knowledge, and without improvement in knowledge about the phenomenon of care, there is no improvement in nursing competencies. Therefore, the second consequence is that there is no improvement in the development of services for citizens. This issue is crucial in our society, as we are witnessing a demographic and epidemiological shift towards ageing and chronicity leading to a greater need for care. All the questions raised are at the origin of this research.

The development of scientific and academic nursing research could be discouraged by the dilemma of publishing to generate an IF and not being read or being read and not generating an IF. For these reasons, the objectives of this study were to identify the habits related to the search, reading, and consultation of the scientific nursing literature and which reasons influence reading, consuming, and publishing in impact journals.

## 2. Materials and Methods

### 2.1. Design

This research consisted of a cross-sectional descriptive study using an ad hoc questionnaire that focused on the uses and habits of scientific reading and production of nurses in Spanish and Portuguese.

### 2.2. Variables

A questionnaire called “Uses and habits of scientific reading and production of nurses in Spanish and Portuguese” (1Q_LcEE-CAPC) was designed based on a literature review [2,7,13] and consensus from a panel of experts. This questionnaire explored which journals are read and why; in which journals publications are published and why; and the applicability of what is researched, published, and read from the point of view of each of the specified profiles. These items, which were transformed into questions, were constructed from the objectives of the study and the concepts underlying the impact factor, such that the relevance for the discipline and the possibility of citations in subsequent studies were assumed. The questionnaires were designed ad hoc; however, their reliability and construct validity were assessed through the factor analysis that can be seen in Table S2. The questions were reviewed by the research team and translated into and cross-culturally adapted to Spanish and Portuguese by bilingual members of the team (Tables S1–S5).

The other variables to be studied were sociodemographic (language, country, sex, experience, age (years), situation (working/studying), public/private work, primary/hospital care, job profile); consulting habits; application; publication; and citation (Table S1).

### 2.3. Study Population and Sample

A multicentre study at the Nuestra Señora de Candelaria University School of Nursing (Spain), University of La Laguna, University of Huelva, University of Alicante, Catholic University of Murcia, Escola Superior de Saúde of Viana do Castelo (Portugal), University of Guadalajara (Mexico), and the Unilab (Brazil) was carried out.

Nurses and nursing students from Spain, Portugal, and Latin America who consented to the use of socio-demographic information and questionnaire responses were selected. Questionnaires that were not completed in their entirety were excluded.

If we assume an infinite sample size (for example, in Spain there are only 200,000 registered nurses) with a confidence level of 95%, a precision of 5%, and a proportion of 50% (to maximise the sample size), the required sample size is 384. Considering Spanish and Portuguese speakers, the required sample would be 768. The total sample obtained was 1042 questionnaires; 30%, that is, 312, of this sample corresponded to the Portuguese-speaking population.

### 2.4. Data Collection and Analysis

The collection period was between January 2021 and August 2021.

Data were collected from the online questionnaire. It was distributed online to nursing students, auxiliary nurses, nurse managers, nurses who are university professors, nursing university professors who are not nurses, nursing university professors who are nurses but work in other professions, and research nurses with publications (Table S1). This population had the necessary tools to access databases in their workplace or study during their research process.

The values taken via the study variables were collected in Excel databases, and a descriptive analysis was carried out. For categorical variables, percentages were calculated. For numerical variables, the measures of centralisation and median were calculated after checking the normality of the obtained data. In this study, the Kolmogorov–Smirnov test allowed for studying if the sample came from a population with a certain distribution (mean and standard deviation) and was not limited to the normal distribution.

Coherence or internal consistency analyses of the data were carried out by using specific tests (Cronbach’s alpha) and factorial validity tests of the questionnaires used (Tables S1–S5). The results were described, and hypothesis testing was carried out in order to identify differentiating variables. In the case of orthogonal factor analysis, it minimised overlapping of these identified aspects.

Finally, a study of correlations between quantitative variables and factor analysis was carried out to group the identified indicators. Results were considered satisfactory when the reliability was 0.8 or higher and when the variance of the factor analysis exceeded 60%.

The Statistical Package for the Social Sciences for Windows (SPSS v.21) (IBM, Armonk, NY, USA), which was licensed by the Research Area of the Nuestra Señora de Candelaria University Hospital, was used to carry out the statistical analysis.

### 2.5. Ethical Considerations

The study was conducted in accordance with the Declaration of Helsinki and approved by the Ethics Committee of the Fundación Instituto Sanitaria de Canarias, Record 19/34. Informed consent was obtained from all subjects involved in the study.

## 3. Results

### 3.1. Sociodemographic Data

The 1Q_LcEE-CAPC questionnaire was sent to a total of 1042 people, of which 417 responded. Of the respondents, 72% were women with a mean age of 37 years and 20 years of experience. Of the sample, 60% came from Spain, 30% from Brazil and Portugal, and 10% from other Latin American countries.

Of the participants, 63% had a healthcare work profile, 80% worked in a public health system, and all of them were almost equally divided between primary and hospital care.

The demographic variables shown in Table 1 were analysed to check their influence on the responses to which journals the respondents read, which ones they referenced, which ones they published in, which ones they knew, and which ones they did not know. To this end, the Fisher’s chi-squared hypothesis test statistic (Table 1) was used for the first 10 results, for each aspect and in Table S4 in full. In these supplementary tables, the journals for which significance was found for any of the explored variables are shown. In general, all variables showed statistically significant differences in this regard.

### 3.2. Preferred Journals for Consuming and Publishing according to Sample Characteristics

In the second part of the questionnaire, for the journals selected in Spanish, Portuguese, and English, there were questions about which journals were read, which journals were referenced, in which journals respondents chose to publish, which journals were known, and which journals were not known. The overall response reliability for these questions was 0.97. The disaggregated reliability analysis for each of them revealed that the lowest reliability found was 0.73 for publishing in Spanish and Portuguese, and the highest was 0.98 for not knowing journals in English (Table 2).

### 3.3. Reasons for Reading Journals

A total of 26 journals in Spanish, 22 journals in Portuguese, and 33 journals in English (Table S1) were included. These journals were selected from the journals indexed in the JCR [3] and SJR [4] indexes and the CINAHL [9] and CUIDEN [10] databases. Table 3 shows the results for each of the variables, which are ordered by frequency of response. Thus, the first six reasons exceeded 250 responses (slightly more than half of the respondents). The reasons for reading according to this were as follows: that the language was understood; for learning and applying what was learnt; that the journal was of open access; for elaborating protocols and work procedures; and that the journal was indexed in scientific databases and in nursing databases. The reliability found for the responses to this questionnaire was 0.75.

The factor analysis on reasons for reading journals revealed the existence of several factors (Table S2). Factors 1 and 2 account for 35% of the total variance. Factor 1, which accounted for 24% of the variance, included being of open access, having peer review, and being indexed in scientific databases and in nursing databases. Factor 2 accounted for 11% of the variance and included understanding the language, learning and applying what was learnt, developing protocols and work procedures, and discussing concepts with peers (Tables S1–S5).

### 3.4. Most Relevant Journals for Reading, Publishing, and Referencing, Known and Not-Known

The responses to these five aspects are shown in Table 4 for the top 15 results for each aspect and in total (Table S3). The most read journals, which exceeded 30%, were Index de Enfermería, Enfermería Clínica, Rol de Enfermería, and the International Journal of Nursing Studies. In Spanish, in addition to the first three, Investigación y Educación en Enfermería and Metas de Enfermería were added at 30%. In Portuguese, none of them exceeded 30%, and in English, only the International Journal of Nursing Studies did so. As for the referenced journals, only Index de Enfermería reached 20.4% of the responses. All of the others were below this percentage.

For the question about in which journals a publication was published, no journal reached 10% of the responses. The top three journals were Enfermería Clínica, Rol de Enfermería, and Revista de Enfermagem UFPE On Line. The first journal for publication in English was the International Journal of Mental Health Nursing.

Table 4 also shows the results for the journals that were known. The only journal that exceeded 30% was the International Journal of Nursing Studies. There were 15 journals that exceeded 20%, of which 9 were in Spanish, 5 in Portuguese, and 1 in English. The results of the question about which journals were unknown are shown in an inverted way, i.e., showing the least unknown journals first in order to visually display coherence with the previous question. There were 20 unknown journals for more than 60% of the answers.

### 3.5. Impact and Variability Indexes according to the Reading, Referencing, Publishing, Knowing, and Not Knowing Criteria

Table 5 provides three examples of the creation of impact indexes from the selected journals according to the criteria included in this study (full analysis in Table S5). The analysis shows the complexity in the combination of the criteria and how it changes the position of the journal in the created ranking (Ranking 1: Most read journals; Ranking 2: Combination of readings, publications, and times referenced; Ranking 3: Known or not known; Ranking 4: Ranking 2 + Ranking 3). As can be seen, the variability that occurs is very important, which highlights the possibility that something similar can occur in the construction of other impact factors.

## 4. Discussion

The results of the study can be summarised as follows: on the one hand, the reasons for reading, using, and publishing in journals are related to language knowledge and to the usefulness for learning and applying knowledge; and on the other hand, open-access journals are read to be used to develop protocols or work procedures and because they are indexed in scientific databases and in nursing databases. In summary, it is possible to state that research journals are read to improve competence in care.

However, the results of the study show that the habits of reading, researching, publishing, and translating care into clinical practice do not correspond to the journals classified in the JCR and SJR. Whether journals are read, referenced, and published in and whether they are known or not known are more influenced by availability and context (age, sex, experience, field of work, etc.) than by impact factor (measured quality) of the journals. This seems to confirm the inadequacy of the IF in the field of nursing, and its impact on nursing clinical practice, skills management, university teaching practice, and access to research funding. The care-focused journals that nurses read, reference, publish in, and know about are not present in impact factor rankings.

Including students in the sample may raise doubts about the rigour of the responses. On the contrary, the research group considered that the participation of students enriched the sample and gave greater quality to the responses. Students consult academic sources, books, and journals upon the recommendation of their professors. In this sense, the inclusion of a group of students in the sample could lead to a slight over-representation of journals recommended by academics. Following this line of argument, we could think that these journals, which are recommended and used by students, are typically used by universities and by their professors and researchers, so they could be considered to be quality journals in the opinion of these academic professionals. In the literature review conducted in preparation for this study, no research studies were identified that specified the presence of nursing publications in journals indexed in JCR and SRJ. Although several authors refer to this lack of research [11,12,13], at the end of this study, no studies were found that attempt to explain this absence. As can be seen in Table 5, variability is a fact which is very important to consider, and which can occur in the construction of other impact factors.

Nursing efforts to address the health threats of infectious diseases and pandemics are becoming increasingly important worldwide. A recent review has shown that global nursing shows a tendency to focus on environmental health and safety, thus reflecting the current interest in the environment worldwide in most of the journals [14]. This is only an example. In the last decade, both the excellence and the universality of mainstream journals have been called into question, with IF [15,16] being considered the only instrument of prestige. The academic value of scientific work has been relegated to the background with the use and abuse of IF metrics, revealing the purely commercial interest of large publishers who impose commercial obstacles and interests on topics to the global conversation on science. China is an example of the shift towards the forced internalisation of science in order to mitigate the distortions it has produced in interactions between science and society [11].

The establishment of institutions of scientific imperialism entails several negative trends: the slowdown of article publication, the loss of a national research culture, and the formulation and presentation of research results to meet the requirements of database indexing. There is a need to rethink possible approaches to mitigate the negative effects associated with the expansion of institutions of scientific imperialism [12]. Some of these negative effects that have already been described are:

- These databases cover mainly journal articles, leaving out other types of documents such as monographs, conference publications, or collective books, which are important for the dissemination and transfer of research.
- A lack of comparability between the impact factors obtained in the different scientific areas, in which consulting and publication habits, as well as the number of journals indexed in each of them in these databases, are different.
- Generating a tendency to self-citation, even if not justified, in order to increase the number of citations.
- Ascribing the same value to any received citation regardless of the journal in which the article was published and even if the article has not been cited. This is because the JCR and SJR are established on the basis of the total number of citations received by the journal in a given period of time.
- Bias with respect to language or country of publication. For example, it is an accepted norm that the title, keywords, and abstract of articles are written in English, regardless of the language in which the journal is published. This might be due to “national literature tradition”, such that authors publish in their mother tongue [8]. On the other hand, the IF of English-language journals is higher than that of multilingual journals, and the IF of multilingual journals becomes higher as the percentage of articles published in English in them increases [15].
- Pressure on researchers to publish in journals included in these rankings. Several studies have identified possible psychopathological disorders in the scientific community related to this issue [17,18].
- Predatory journals in which the profit, given the low visibility of the journal, comes from the editors themselves. There are journals that contact authors to ask them to publish in them, edit the work, and pay for the publication. They have no peer review or other guarantee for authors of error-free publication, but only an ISBN or ISSN to justify publication [19].
- Open access [20]: there are journals that are published in an open system, free of charge. Originally, the aim of this type of publication was to make research results available to the scientific community, as access to published articles is often paid for. However, publishing in these open-access journals has an added cost to the effort of researching and writing the research article. The fee for peer review can range from €600 to €6000 per article; in many cases, the researcher wonders whether they can afford it. For this reason, some competitive research calls already include open-access publication as one of the budgetary options. However, if the researcher cannot access a call, or is not awarded a grant, this option is not made available. The alternative in these cases for researchers is to resort to anonymous patrons or personal payment for their publications in these journals.

In the case of open access, payment for publication creates a paradox. If a researcher publishes work funded by a competitive call for papers in a journal with a high IF, the dissemination of the research results is paid for at least two or three times: the first time, through the grant; the second time, from the subscription to the publication, given that the corresponding health service has subsidised the research; and then, the institution to which the researcher belongs has to pay for institutional access.

Furthermore, according to the European initiative “cOAlition S” [20] from 2021, all academic publications on research results that have been subsidised with public money or privately provided by national, regional, and international research councils and funding bodies must be published in open-access journals, which are open to everyone and available for free scientific intellectual exchange. So far, no Spanish institution has joined this initiative.

- Invisibility of scientific production in one’s own language. Most researchers cite the majority of studies on a topic in English to the detriment of their own colleagues’ published research, in this case, in Spanish [21]. This is compounded by the difficulty of translation into English. Due to this fact, non-English speaking authors may require the use of translation services or at least grammatical and semantic revision of the English language. The cost of these services is sometimes very high.
- Saturation of journal articles. Publishers such as SAGE [22] explicitly report high acceptance rates due to saturation. It also happens that some open-access journals exclude for review approximately 50% of the articles they receive.

Finally, the DORA statement [23] declares that “the IF is frequently used as the primary parameter to compare the scientific output of individuals and institutions. The IF, now published by Clarivate Analytics [3], was originally created as a tool to help librarians identify journals to purchase, not as a measure of the scientific quality of research in an article”.

With this in mind, it is critical to understand that the impact factor has a number of well-documented shortcomings as a tool for research evaluation, partially due to the disproportionate use of it. The active promotion and safeguarding of the integrity of the editing and publication process may help to improve the scientific influence of academic journals, which in itself is the cornerstone of sustaining the significance of the impact factor [24].

### Limitations

The study conducted presents some limitations related to the composition of the sample, especially with regard to its student profile, which consists of mostly Portuguese speakers. Moreover, with 60% of the sample being from Spain, 30% from Brazil and Portugal, and 10% from other Latin American countries, this study should be considered an approximate view. Following this reasoning, our investigation has faced a series of limitations related to the collection of data on the IF of journals, which have already been referenced, and which could be summarised in the following statements: citation distribution within journals is highly skewed [1,2,12,21,25,26,27,28,29,30]; the properties of the impact factor are field-specific: it is a composite of multiple, highly diverse article types, including primary research papers and reviews [1,10,12,25,26,27,28,29,30]; impact factors can be manipulated (or evaluated) by editorial policy [7,12,25,26,27,28,29,30]; and the data used to calculate the impact factor is neither transparent nor openly available to the public [5,10,11,12,13,16,17,18,19,20,22,23,25,26,27,28].

## 5. Conclusions

The research, reading, and publishing habits of nurses and nursing students as applied to impact journals show that the reasons for reading, consuming, and publishing in journals are related to language proficiency, usefulness for the learning process, and for knowing how to apply specific knowledge. Open-access journals are used to develop protocols and are often indexed in scientific and nursing databases. However, it should be noted that reading, research, and publication habits are not always associated with journals indexed in JCR and SJR. In this case, the availability of information and the context of the researcher (age, sex, experience, field of work, etc.) seem to be more influential. From these results, it can be concluded that an index specific to care research publications and/or nursing publications should be created. This would positively impact the scientific production of care, giving greater visibility and transferability to the results obtained. Consequently, there would be an impact on society and on the quality of the professional care received. The results of this research imply that reading research journals is recommended in order to increase competence in healthcare.

## Figures and Tables

**Table 1:** Characteristics of the sample responding to the 1Q_LcEE-CAPC Questionnaire.

	% (*n*)	Categories
Sex	71.9% (299)	Female
	27.4% (114)	Male
	0.7% (4)	Rather not say
	100.0% (417)	Total
Age	37.51 years	Mean
	37.00 years	Median
	14.30 years	Standard deviation
Age percentiles	27.8%	Up to 24 years
	22.8%	Between 24 and 37 years
	25.1%	Between 37 and 49 years
	24.3%	More than 49 years
Professional Experience	20.54 years	Mean
	20.00 years	Median
	12.05 years	Standard deviation
Professional Experience Percentiles	23.2%	Student
	2.7%	1–2 years
	24.3%	2–14 years
	26%	14–28 years
	23.8%	More than 28 years
Countries	60.7%	Spain
	19.3%	Brazil
	10.7%	Portugal
	7.1%	Mexico
	2.4%	Other
Worker profile	63%	Auxiliary
	48.4%	Exclusively auxiliary
	39.5%	Professor
	19.7%	Exclusively professor
	20.7%	Researcher
	3.3%	Exclusively researcher
	12.7%	Manager
	3.3%	Exclusively manager
Place of work Public–Private	80.2%	Public institution
	15.9%	Private institution
	4%	Both
Place of work Primary care–Hospital care	54.9%	Primary care
	42.9%	Hospital care
	2.3%	Both

**Table 2:** 1Q_LcEE-CAPC Questionnaire. Contrast of hypotheses, demographic variables, and responses to “knowing and not knowing the top 10 journals in Spanish, Portuguese, and English”. Source: self-elaborated.

Reading Journals	Language	Country	Sex	Experience	Age (Years)	Situation Working/Studying	Public/Private Work	Primary/Hospital Care	Job Profile
Cultura de los Cuidados	0.000	0.000	0.027	0.000	0.000	0.000	0.129	0.208	0.012
Enfermería Clínica	0.000	0.000	0.003	0.000	0.000	0.000	0.016	0.095	0.003
Enfermería Comunitaria	0.000	0.000	0.014	0.000	0.000	0.000	0.074	0.000	0.000
Enfermería Global	0.047	0.190	0.890	0.305	0.070	0.017	0.007	0.330	0.000
Enfermería Intensiva	0.021	0.081	0.001	0.309	0.292	0.050	0.079	0.293	0.012
Index de Enfermería	0.000	0.000	0.072	0.013	0.004	0.000	0.030	0.461	0.011
International Journal of Nursing Studies	0.028	0.107	0.247	0.026	0.602	0.125	0.060	0.646	0.036
Investigación y Educación en Enfermería	0.479	0.484	0.144	0.223	0.774	0.077	0.165	0.631	0.033
Journal of Advanced Nursing	0.027	0.106	0.517	0.016	0.040	0.001	0.019	0.547	0.000
Metas de Enfermería	0.000	0.000	0.005	0.000	0.000	0.000	0.002	0.000	0.000
Revista Cubana de Enfermería	0.001	0.013	0.671	0.357	0.479	0.037	0.072	0.819	0.148
Revista CUIDARTE	0.001	0.020	0.494	0.019	0.068	0.660	0.488	0.306	0.258
Revista ENE de Enfermería	0.000	0.000	0.047	0.001	0.000	0.000	0.017	0.010	0.000
Revista Latino-Americana de Enfermagem	0.000	0.000	0.921	0.830	0.917	0.735	0.521	0.020	0.002
Revista Rol de Enfermería	0.000	0.000	0.000	0.000	0.000	0.000	0.015	0.000	0.000
**Referencing journals**									
Brasileira de Enfermagem	0.000	0.000	0.378	0.168	0.117	0.003	0.489	0.002	0.001
Enfermería Clínica	0.000	0.002	0.351	0.002	0.017	0.008	0.009	0.191	0.000
Enfermería Comunitaria	0.000	0.088	0.463	0.276	0.296	0.193	0.026	0.562	0.848
Enfermería Docente	0.001	0.512	0.152	0.114	0.818	0.646	0.354	0.000	0.000
Enfermería Global	0.259	0.527	0.628	0.795	0.528	0.274	0.260	0.045	0.000
Escola Anna Nery Revista de Enfermagem	0.000	0.000	0.810	0.094	0.218	0.005	0.680	0.177	0.000
Index de Enfermería	0.000	0.001	0.045	0.038	0.038	0.037	0.187	0.954	0.000
International Journal of Nursing Studies	0.031	0.305	0.565	0.089	0.428	0.995	0.060	0.573	0.139
Journal of Advanced Nursing	0.081	0.490	0.641	0.084	0.067	0.003	0.008	0.461	0.002
Metas de Enfermería	0.000	0.000	0.368	0.001	0.000	0.000	0.013	0.246	0.000
Revista Cubana de Enfermería	0.001	0.364	0.185	0.405	0.875	0.804	0.405	0.325	0.003
Revista da Escola de Enfermagem da USP	0.000	0.000	0.784	0.682	0.616	0.870	0.146	0.260	0.000
Revista Latino-Americana de Enfermagem	0.000	0.000	0.746	0.242	0.065	0.233	0.259	0.168	0.006
Revista Rol de Enfermería	0.000	0.001	0.233	0.013	0.003	0.007	0.263	0.043	0.001
Texto & Contexto: Enfermagem	0.000	0.000	0.250	0.919	0.338	0.892	0.341	0.006	0.000
**Publishing in journals**									
Brasileira de Enfermagem	0.000	0.000	0.093	0.010	0.029	0.009	0.011	0.247	0.000
Cultura de los Cuidados	0.036	0.367	0.093	0.001	0.021	0.008	0.096	0.411	0.000
Enfermería Clínica	0.023	0.267	0.034	0.005	0.002	0.000	0.001	0.832	0.850
Enfermería Global	0.247	0.085	0.799	0.137	0.040	0.002	0.240	0.083	0.000
Enfermería Intensiva	0.152	0.599	0.387	0.466	0.543	0.074	0.228	0.107	0.011
Index de Enfermería	0.962	0.998	0.025	0.003	0.037	0.019	0.252	0.497	0.001
International Journal of Mental Health Nursing	0.000	0.000	0.029	0.252	0.464	0.015	0.568	0.060	0.682
Metas de Enfermería	0.040	0.366	0.252	0.008	0.058	0.003	0.764	0.891	0.000
Revista Baiana de Enfermagem	0.000	0.000	0.002	0.254	0.197	0.074	0.148	0.758	0.000
Revista da Escola de Enfermagem da USP	0.000	0.006	0.257	0.457	0.089	0.009	0.042	0.247	0.000
Revista da Rede de Enfermagem do Nordeste	0.000	0.000	0.425	0.010	0.024	0.004	0.007	0.331	0.000
Revista de Enfermagem UFPE On Line	0.000	0.000	0.907	0.009	0.068	0.263	0.026	0.278	0.000
Revista ENE de Enfermería	0.026	0.009	0.511	0.000	0.000	0.002	0.007	0.432	0.056
Revista Latino-Americana de Enfermagem	0.000	0.004	0.753	0.160	0.083	0.004	0.042	0.427	0.000
Revista Rol de Enfermería	0.015	0.208	0.150	0.000	0.000	0.001	0.349	0.202	0.000
**Knowing journals**									
Aquichán	0.085	0.129	0.020	0.087	0.002	0.003	0.012	0.200	0.000
Avances en Enfermería	0.001	0.027	0.003	0.085	0.020	0.003	0.002	0.008	0.010
Cultura de los Cuidados	0.004	0.029	0.007	0.129	0.071	0.025	0.128	0.001	0.200
Enfermería Clínica	0.000	0.010	0.021	0.012	0.000	0.001	0.017	0.047	0.032
Enfermería Global	0.011	0.076	0.005	0.013	0.021	0.012	0.046	0.166	0.083
Enfermería Intensiva	0.002	0.049	0.045	0.064	0.006	0.009	0.103	0.319	0.001
Enfermería Nefrológica	0.000	0.000	0.091	0.008	0.008	0.000	0.090	0.010	0.001
Enfermería Universitaria	0.039	0.345	0.759	0.620	0.545	0.601	0.754	0.653	0.443
Gerokomos	0.000	0.000	0.007	0.001	0.000	0.000	0.000	0.013	0.000
Index de Enfermería	0.000	0.000	0.043	0.245	0.333	0.720	0.104	0.111	0.218
Investigación en Enfermería: imagen y desarrollo	0.153	0.162	0.478	0.142	0.070	0.874	0.969	0.028	0.776
Investigación y Educación en Enfermería	0.007	0.048	0.216	0.977	0.862	0.391	0.815	0.004	0.008
Revista CUIDARTE	0.029	0.222	0.346	0.641	0.497	0.043	0.221	0.047	0.135
Revista ENE de Enfermería	0.000	0.001	0.061	0.195	0.027	0.049	0.640	0.010	0.000
Temperamentvm	0.007	0.130	0.037	0.048	0.006	0.003	0.017	0.632	0.040
**Not knowing journals**									
American Journal of Critical Care	0.069	0.186	0.861	0.117	0.057	0.095	0.141	0.175	0.024
Avances en Enfermería	0.000	0.000	0.510	0.002	0.000	0.000	0.005	0.302	0.018
Diabetes Care	0.155	0.255	0.813	0.756	0.435	0.826	0.578	0.518	0.308
Enfermería Clínica	0.000	0.000	0.606	0.000	0.000	0.000	0.040	0.154	0.005
Enfermería Comunitaria	0.000	0.000	0.183	0.000	0.000	0.000	0.037	0.001	0.005
Enfermería Global	0.000	0.000	0.379	0.049	0.006	0.000	0.064	0.392	0.002
Enfermería Intensiva	0.000	0.002	0.140	0.006	0.000	0.000	0.582	0.773	0.001
European Journal of Cardiovascular Nursing	0.033	0.109	0.179	0.488	0.074	0.293	0.476	0.184	0.039
International Journal of Mental Health Nursing	0.612	0.623	0.333	0.676	0.337	0.674	0.739	0.044	0.165
International Journal of Nursing Studies	0.076	0.070	0.227	0.018	0.138	0.560	0.388	0.893	0.381
Investigación y Educación en Enfermería	0.039	0.187	0.462	0.786	0.467	0.010	0.836	0.434	0.198
Journal of Advanced Nursing	0.003	0.030	0.954	0.384	0.268	0.007	0.228	0.192	0.003
Revista CUIDARTE	0.001	0.005	0.991	0.042	0.024	0.000	0.547	0.047	0.084
Revista ENE de Enfermería	0.000	0.000	0.203	0.020	0.002	0.000	0.288	0.230	0.001
Revista Rol de Enfermería	0.000	0.000	0.063	0.000	0.000	0.000	0.005	0.000	0.000

**Table 3:** 1Q_LcEE-CAPC Questionnaire. Reasons for reading journals, results, and reliability. Source: self-elaborated.

Reasons	Spanish	Portuguese	English	Total
I understand the language	240	131	110	481
I learn and apply what I have learnt	155	87	107	349
It is open access	153	95	76	324
I develop protocols, procedures, work	122	89	108	319
It is indexed in scientific databases	125	93	99	317
It is indexed in nursing databases	107	76	67	250
It has an impact factor	53	45	82	180
I discuss with my colleagues	72	34	72	178
I have it available in my institution	76	44	54	174
It has peer review	42	42	55	139
It is disseminated on social networks	53	23	30	106
The publisher has prestige	39	22	39	100
The abstract is in other languages	30	33	36	99
I have an individual subscription	24	1	16	41

Reliability, Cronbach’s alpha: 0.751.

**Table 4:** 1Q_LcEE-CAPC Questionnaire. Responses to “reading, referencing, publishing in, and knowing or not knowing journals in Spanish, Portuguese, and English”.

Reading		Referencing		Publishing		Knowing		Not Knowing	
Journals	%/Total	Journals	%/Total	Journals	%/Total	Journals	%/Total	Journals	%/Total
Cultura de los Cuidados	22.1% 87	Brasileira de Enfermagem	14.0% 55	Brasileira de Enfermagem	4.6% 18	Aquichán	9.1% 36	American Journal of Critical Care	40.9% 161
Enfermería Clínica	37.6% 148	Enfermería Clínica	19.5% 77	Cultura de los Cuidados	3.3% 13	Avances en Enfermería	22.3% 88	Avances en Enfermería	42.6% 168
Enfermería Comunitaria.	29.7% 117	Enfermería Comunitaria.	29.7% 117	Enfermería Clínica	7.9% 31	Cultura de los Cuidados	19% 75	Diabetes Care	39.8% 157
Enfermería Global	29.7% 117	Enfermería Docente	12.2% 48	Enfermería Global	4.6% 18	Enfermería Clínica	25.1% 99	Enfermería Clínica	31.5% 124
Enfermería Intensiva	23.4% 92	Enfermería Global	17.8% 70	Enfermería Intensiva	2.8% 11	Enfermería Global	23.1% 91	Enfermería Comunitaria.	38.1% 150
Index de Enfermería	45.7% 180	Escola Anna Nery Revista de Enfermagem	8.1% 32	Index de Enfermería	5.1% 20	Enfermería Intensiva	32.1% 91	Enfermería Global	36% 142
International Journal of Nursing Studies	32.5% 128	Index de Enfermería	21.1% 83	International Journal of Mental Health Nursing	4.1% 16	Enfermería Nefrológica	19.8% 78	Enfermería Intensiva	40.6% 160
Investigación y Educación en Enfermería	32% 126	International Journal of Nursing Studies	16.2% 64	Metas de Enfermería	5.3% 21	Enfermería universitaria	14.2% 56	European Journal of Cardiovascular Nursing	40.9% 161
Journal of Advanced Nursing	23.4% 92	Journal of Advanced Nursing	14.5% 57	Revista Baiana de Enfermagem	2.8% 11	Gerokomos	15.2% 60	International Journal of Mental Health Nursing	41.4% 163
Metas de Enfermería	31.0% 122	Metas de Enfermería	31.0% 122	Revista da Escola de Enfermagem da USP	4.6% 18	Index de Enfermería	27.7% 109	International Journal of Nursing Studies	22.8% 90
Revista Cubana de Enfermería	21.3% 84	Revista Cubana de Enfermería	21.3% 84	Revista da Rede de Enfermagem do Nordeste	3.8% 15	Investigación en Enfermería: imagen y desarrollo	13.2% 52	Investigación y Educación en Enfermería	31.5% 124
Revista CUIDARTE	25.1% 99	Revista da Escola de Enfermagem da USP	13.2% 52	Revista de Enfermagem UFPE On Line	6.1% 24	Investigación y Educación en Enfermería	23.1% 91	Journal of Advanced Nursing	34.3% 135
Revista ENE de Enfermería	25.4% 100	Revista Latino-Americana de Enfermagem	14.0% 55	Revista ENE de Enfermería	4.6% 18	Revista CUIDARTE	28.9% 114	Revista CUIDARTE	33.2% 131
Revista Latino-Americana de Enfermagem	23.6% 93	Revista Rol de Enfermería	34.3% 135	Revista Latino-Americana de Enfermagem	3.8% 15	Revista ENE de Enfermería	21.8% 86	Revista ENE de Enfermería	42.1% 166
Revista Rol de Enfermería	34.3% 135	Texto & Contexto: Enfermagem	13.2% 52	Revista Rol de Enfermería	6.1% 24	Temperamentvm	8.6% 34	Revista Rol de Enfermería	35.8% 141

**Table 5:** Impact and variability indexes according to the reading, referencing, publishing in, knowing, and not knowing criteria of the top 15 journals in Spanish, Portuguese, and English.

Journals	Rank 1. Total	Rank 2. Reading Referencing Publishing	Rank 3. Knowing Not Knowing	Rank 4. Reading
Cultura de los Cuidados	14	26	48	18
Enfermería Clínica	2	43	74	3
Enfermería Comunitaria	8	21	37	11
Enfermería Global	7	31	45	9
Enfermería Intensiva	12	79	35	5
Index de Enfermería	1	59	54	2
International Journal of Nursing Studies	4	37	39	6
Investigación y Educación en Enfermería	5	23	59	13
Journal of Advanced Nursing	13	9	44	17
Metas de Enfermería	6	46	53	8
Revista Cubana de Enfermería	15	82	82	1
Revista CUIDARTE	10	6	41	16
Revista ENE de Enfermería	9	80	81	4
Revista Latino-Americana de Enfermagem	11	41	51	12
Revista Rol de Enfermería	3	38	40	7

## Data Availability

Data are available on request from the authors.

## Supplementary Materials

The following supporting information can be downloaded at: https://www.mdpi.com/article/10.3390/ijerph20064697/s1, Table S1: Questionnaires: Uses and habits of scientific reading and production of nurses in Spanish and Portuguese; Table S2: 1Q_LcEE-CAPC Questionnaire factor analysis. Reasons for reading journals; Table S3: 1Q_LcEE-CAPC Questionnaire. Contrast of hypothesis, demographic variables, and responses to reading, referencing, publishing, knowing, and not knowing journals in Spanish, Portuguese, and English; Table S4: 1Q_LcEE-CAPC Questionnaire. Responses to “reading, referencing, publishing in, knowing, and not knowing journals in Spanish, Portuguese, and English”; Table S5: Analysis for the creation of impact indexes and their variability according to the included criteria.

## References

[1] De Solla Price D.J. (2016). A general theory of bibliometric and other cumulative advantage processes. J. Am. Soc. Inf. Sci..

[2] Lima-Serrano M., Lima-Rodríguez J.S., Porcel-Gálvez A.M., Gil-García E. (2015). ¿Cómo mejorar la visibilidad de la investigación enfermera española? Publicaciones de referencia e índices de calidad. [How to improve the visibility of Spanish research in nursing? Reference journals and quality indexes]. Enferm. Clin..

[3] Clarivate Analytics (2022). Web of Science Confident Research Begins Here. https://clarivate.com/webofsciencegroup/solutions/web-of-science/.

[4] Elsevier (2022). Scopus Expertly Curated Abstract & Citation Database. https://www.elsevier.com/solutions/scopus.

[5] Bergan S., Hazelkorn E., Howard L., Huertas E., Marcos S., Martínez I. (2018). Informe Sobre el Estado de la Evaluación Externa de la Calidad de las Universidades Españolas 2017.

[6] Ministerio de Ciencia, Innovación y Universidades (2018). Resolución de 28 de Diciembre de 2018 de la Directora del Instituto de Salud Carlos III por la que se Aprueba la Convocatoria Correspondiente al año 2019 Mediante Tramitación Anticipada de Concesión de Subvenciones de Acción Estratégica en Salud 2017–2020, del Programa Estatal de Investigación Científica y Técnica y de Innovación 2017–2020.

[7] Llauradó-Serra M., Güell-Baró R., Castanera-Duro A., Sandalinas I., Argilaga E., Fortes-del Valle M.L. (2016). Barreras y motivaciones de los profesionales de enfermería para la utilización de la investigación en Unidades de Cuidados Intensivos y en el Servicio de Emergencias Médicas. Enferm. Intensiv..

[8] Morales Asencio J.M., Hueso Montoro C., de Pedro-Gómez J.E., Bennasar-Veny M. (2017). 1977–2017: La investigación enfermera en España tras 40 años en la Universidad. Enferm. Clin..

[9] EBSCO: CINAHL Database® (2022). The Cumulative Index to Nursing and Allied Health Literature. https://health.ebsco.com/products/the-cinahl-database.

[10] (2022). Fundación Index: CUIDEN Base de datos Bibliográfica Sobre Cuidados de Salud en Iberoamérica^®^. Fondo de Publicaciones Periódicas de la Fundación Index: CATÁLOGO REHIC. http://www.fundacionindex.com/bibliometria/listado-rehic.php.

[11] Popov Y.V., Popova N.G., Kochetkov D.M. (2021). On “Scientific Imperialism”. Russ. Soc. Sci. Rev..

[12] Adler R., Ewing J., Taylor P. (2009). Citation Statistics: A Report from the International Mathematical Union (IMU) in Cooperation with the International Council of Industrial and Applied Mathematics (ICIAM) and the Institute of Mathematical Statistics (IMS). Stat. Sci..

[13] Cidoncha-Moreno M.A., Ruíz de Alegría-Fernández de Retanab B. (2017). Percepción de barreras para la utilización de la investigación en enfermeras de Osakidetza. Enferm. Clin..

[14] Yatsu H., Saeki A. (2022). Current trends in global nursing: A scoping review. Nurs. Open.

[15] Abad J.C.S., Alencar R.M., Marimon B.H., Marimon B., Silva A.C.C., Jancoski H., Rezende R.S., Alves-Silva E. (2020). Publishing in English is associated with an increase of the impact factor of Brazilian biodiversity journals. An. Acad. Bras. Cienc..

[16] Beigel F. (2021). Proteger las Revistas Nacionales de Calidad para Revalorizar la Producción Científica con Relevancia Local. Consejo Latinoamericano de Ciencias Sociales (CLACSO). https://www.clacso.org/proteger-las-revistas-nacionales-de-calidad-para-revalorizar-la-produccion-cientifica-con-relevancia-local/.

[17] Buela-Casal G. (2003). Evaluación de la calidad de los artículos y de las revistas científicas: Propuesta del factor de impacto ponderado y de un índice de calidad. Psicothema.

[18] Barria-Traverso D., González-Bustamante B., Cisternas Guasch C. (2019). La literatura sobre gobierno abierto en español. Análisis sobre las dinámicas de producción y citación. Nóesis Rev. Cienc. Soc. Humanid..

[19] Jiménez-Contreras E., Jiménez-Segura J.J. (2016). Las revistas depredadoras, una nueva epidemia científica. CYF.

[20] European Science Foundation (2022). Making Full and Immediate Open Access a Reality. https://www.coalition-s.org/.

[21] Díaz-Membrivesa M., Farrero-Muñoz S., Lluch-Canut M.T. (2012). Características de las Publicaciones Enfermeras en Revistas con Factor de Impacto [Characteristics of Nursing Publications in Journals with Impact Factor]. Enferm. Clin..

[22] SAGE Publishing (2022). https://uk.sagepub.com/en-gb/eur/your-paper-and-peer-review.

[23] San Francisco Declaration on Research Assessment (DORA) (2022). Declaración Sobre la Evaluación de la Investigación de San Francisco (DORA). https://sfdora.org/.

[24] Wang J.L., Li X., Fan J.R., Yan J.P., Gong Z.M., Zhao Y., Wang D.M., Ma L., Ma N., Guo D.M. (2022). Integrity of the editing and publishing process is the basis for improving an academic journal’s Impact Factor. World J. Gastroenterol..

[25] Seglen P.O. (1997). Why the impact factor of journals should not be used for evaluating research. BMJ.

[26] Nature (2005). Not-so-deep impact. Nature.

[27] Vanclay J.K. (2012). Impact Factor: Outdated artefact or stepping-stone to journal certification. Scientometrics.

[28] The PLoS Medicine Editors (2006). The Impact Factor Game. PLoS Med..

[29] Rossner M., Van Epps H., Hill E. (2007). Show me the data. J. Cell Biol..

[30] Rossner M., Van Epps H., Hill E. (2008). Irreproducible results: A response to Thomson Scientific. J. Cell Biol..

