# Supplementary material for: Research, Reading, and Publication Habits of Nurses and Nursing Students Applied to Impact Journals: International Multicentre Study
Source: Int J Environ Res Public Health. 2023 Mar 7;20(6):4697. doi: 10.3390/ijerph20064697 (PMC10049027; doi:10.3390/ijerph20064697)
Supplement: Supplementary file 1 [file ijerph-20-04697-s001.zip › Table S1. 1Q_LcEE-CAPC questionnaire.pdf]

Table S1. Questionnaires: Uses and habits of scientific reading and production of nurses in Spanish and Portuguese.

1Q_LcEE-CAPC for: nursing students, auxiliary nurses, nurse managers, nurses who are university professors, nursing university professors who are not nurses, nursing university professors who are nurses but work in other professions, research nurses with publications.

Dear colleague, thank you for your collaboration in completing this questionnaire. With it, we intend to elaborate a synthetic quality index of nursing publications in scientific journals, with the aim of improving the impact of nursing publications in Spanish and Portuguese. The selected journals are taken from the Cuiden Citation, WoS, and Scopus indexes and occupy the first places in their respective rankings.

It will take you no more than 15 minutes to complete.

Do you agree to answer this survey? [YES] [NO].

1. Sex [1 Female] [2 Male] [3 Rather not say]
2. Age
3. Time worked in years (if less than one, indicate 0)
4. Place of work: [1 Primary Care] [2 Hospital Care] [3 Education] [4 Research] [5 Management] [Student] [7 Others] If you indicated ‘Others’, please, specify.
5. If you are a professor, can you specify? [Nurses who are university professors] [Nursing university professors who are not nurses] [Nursing university professors who are nurses but work in other professions]
6. In the last 5 years, have you read any of these journals? [Index] [Investigación Y Educación En Enfermería] [Aquichán] [Enfermería Intensiva] [Enfermería Universitaria] [Enfermería Global] [Enfermería Nefrológica. Revista Oficial De La Sociedad Española De Enfermería Nefrológica] [Revista ENE De Enfermería] [Revista CUIDARTE] [Avances En Enfermería] [Enfermería Clínica] [Cultura De Los Cuidados] [Investigación En Enfermería: Imagen Y Desarrollo] [Temperamentvm] [Gerokomos] [Revista Ética De Los Cuidados] [Archivos De La Memoria] [Revista Tesela. Revista De La Asociación Nacional De Directivos De Enfermería] [Revista Cubana De Enfermería] [Revista De Enfermería Del Instituto Mexicano Del Seguro Social] [Metas De Enfermería] [Enfermería Comunitaria. Revista Internacional De Cuidados De Salud Familiar Y Comunitaria] [Matronas Profesión] [Revista Rol De Enfermería] [Enfermería Docente] [Biblioteca Lascasas] [Other Journals In Spanish]; [Revista Da Escola De Enfermagem] [Texto & Contexto: Enfermagem] [Escola Anna Nery Revista De Enfermagem] [Revista Latino-Americana De Enfermagem] [Revista Brasileira De Enfermagem] [Revista Gaúcha De Enfermagem] [Acta Paulista De Enfermagem] [Ciência, Cuidado E Saúde]

[Revista Eletrônica De Enfermagem] [Revista De Rede De Enfermagem Do Nordeste] [Cogitare Enfermagem] [Revista De Enfermagem Da UFSM] [Revista Mineira De Enfermagem] [Enfermagem Em Foco] [SOBECC, Em Revista] [Revista De Enfermagem Referência] [Revista De Enfermagem Do Centro-Oeste Mineiro] [Revista De Enfermagem UFPE On Line] [Revista Baiana De Enfermagem] [Revista De Enfermagem Da Universidade Federal Do Piauí] [Revista De Pesquisa: Cuidado É Fundamental. Nursing & Research] [Revista De Enfermagem Atenção E Saùde] [Other Journals In Portuguese]; [International Journal Of Nursing Studies] [Journal Of Nursing Scholarship] [European Journal Of Cardiovascular Nursing] [Nursing Outlook] [European Journal Of Cancer Care] [Birth-Issues In Perinatal Care] [Journal Of Advanced Nursing] [Worldviews On Evidence-Based Nursing] [Journal Of Cardiovascular Nursing] [Nurse Education Today] [American Journal Of Critical Care] [International Journal Of Mental Health Nursing] [Journal Of Family Nursing] [Australian Critical Care] [Journal Of Tissue Viability] [Journal Of Nursing Management] [Nursing Ethics] [Cancer Nursing] [Journal Of Human Lactation] [Women And Birth] [World Psychiatry][Diabetes Care][Stroke][American Journal Of Clinical Nutrition][International Journal Of Obesity][Resuscitation][International Journal Of Behavioral Nutrition And Physical Activity][Nutrition Reviews] [Current Opinion In HIV And AIDS] [Advances In Nutrition] [Journal Of The American Medical Directors Association][Journal Of Pain And Symptom Management][International Journal Of Nursing Studies][Journal Of Palliative Medicine][Journal Of Nursing Scholarship][Nurse Education Today][Nursing Outlook][Patient][Worldviews On Evidence-Based Nursing][Journal Of Advanced Nursing] [Other International Journals]

7. In the last 5 years, have you published in any of these journals? [Index] [Investigación y Educación en Enfermería] [Aquichán] [Enfermería Intensiva] [Enfermería universitaria] [Enfermería Global] [Enfermería Nefrológica. Revista Oficial de la Sociedad Española de Enfermería Nefrológica] [Revista ENE de Enfermería] [Revista CUIDARTE] [Avances en Enfermería] [Enfermería Clínica] [Cultura de los Cuidados] [Investigación en enfermería: imagen y desarrollo] [Temperamentvm] [Gerokomos] [Revista Ética de los Cuidados] [Archivos de la Memoria] [Revista Tesela. Revista de la Asociación Nacional de Directivos de Enfermería] [Revista Cubana de Enfermería] [Revista de Enfermería del Instituto Mexicano del Seguro Social] [Metas de Enfermería] [Enfermería Comunitaria. Revista internacional de cuidados de salud familiar y comunitaria] [Matronas Profesión] [Revista Rol de Enfermería] [Enfermería Docente] [Biblioteca Lascasas] [Other journals in Spanish]; [Revista da Escola de Enfermagem] [Texto & Contexto: Enfermagem] [Escola Anna Nery Revista de Enfermagem] [Revista Latino-Americana de Enfermagem] [Revista Brasileira de Enfermagem] [Revista Gaúcha de

Enfermagem] [Acta Paulista de Enfermagem] [Ciência, Cuidado e Saúde] [Revista Eletrônica de Enfermagem] [Revista de Rede de Enfermagem do Nordeste] [Cogitare Enfermagem] [Revista de Enfermagem da UFSM] [Revista Mineira de Enfermagem] [Enfermagem em Foco] [SOBECC, em Revista] [Revista de Enfermagem Referência] [Revista de Enfermagem do Centro-Oeste Mineiro] [Revista de Enfermagem UFPE On Line] [Revista Baiana de Enfermagem] [Revista de Enfermagem da Universidade Federal do Piauí] [Revista de Pesquisa: Cuidado é Fundamental. Nursing & Research] [Revista de Enfermagem Atenção e Saùde] [Other journals in Portuguese]; [International Journal Of Nursing Studies] [Journal Of Nursing Scholarship] [European Journal Of Cardiovascular Nursing] [Nursing Outlook] [European Journal Of Cancer Care] [Birth-Issues In Perinatal Care] [Journal Of Advanced Nursing] [Worldviews On Evidence-Based Nursing] [Journal Of Cardiovascular Nursing] [Nurse Education Today] [American Journal Of Critical Care] [International Journal Of Mental Health Nursing] [Journal Of Family Nursing] [Australian Critical Care] [Journal Of Tissue Viability] [Journal Of Nursing Management] [Nursing Ethics] [Cancer Nursing] [Journal Of Human Lactation] [Women and Birth] [World Psychiatry][Diabetes Care][Stroke][American Journal of Clinical Nutrition][International Journal of Obesity][Resuscitation][International Journal of Behavioral Nutrition and Physical Activity][Nutrition Reviews] [Current Opinion in HIV and AIDS] [Advances in Nutrition] [Journal of the American Medical Directors Association][Journal of Pain and Symptom Management][International Journal of Nursing Studies][Journal of Palliative Medicine][Journal of Nursing Scholarship][Nurse Education Today][Nursing Outlook][Patient][Worldviews on Evidence-Based Nursing][Journal of Advanced Nursing] [other international journals]

8. In the last 5 years, have you referenced any article from these journals? [Index] [Investigación y Educación en Enfermería] [Aquichán] [Enfermería Intensiva] [Enfermería universitaria] [Enfermería Global] [Enfermería Nefrológica. Revista Oficial de la Sociedad Española de Enfermería Nefrológica] [Revista ENE de Enfermería] [Revista CUIDARTE] [Avances en Enfermería] [Enfermería Clínica] [Cultura de los Cuidados] [Investigación en enfermería: imagen y desarrollo] [Temperamentvm] [Gerokomos] [Revista Ética de los Cuidados] [Archivos de la Memoria] [Revista Tesela. Revista de la Asociación Nacional de Directivos de Enfermería] [Revista Cubana de Enfermería] [Revista de Enfermería del Instituto Mexicano del Seguro Social] [Metas de Enfermería] [Enfermería Comunitaria. Revista internacional de cuidados de salud familiar y comunitaria] [Matronas Profesión] [Revista Rol de Enfermería] [Enfermería Docente] [Biblioteca Lascasas] [Other journals in Spanish]; [Revista da Escola de Enfermagem] [Texto & Contexto: Enfermagem] [Escola Anna Nery Revista de

Enfermagem] [Revista Latino-Americana de Enfermagem] [Revista Brasileira de Enfermagem] [Revista Gaúcha de Enfermagem] [ACTA Paulista de Enfermagem] [Ciência, Cuidado e Saúde] [Revista Eletrônica de Enfermagem] [Revista de Rede de Enfermagem do Nordeste] [Cogitare Enfermagem] [Revista de Enfermagem da UFSM] [Revista Mineira de Enfermagem] [Enfermagem em Foco] [SOBECC, em Revista] [Revista de Enfermagem Referência] [Revista de Enfermagem do Centro-Oeste Mineiro] [Revista de Enfermagem UFPE On Line] [Revista Baiana de Enfermagem] [Revista de Enfermagem da Universidade Federal do Piauí] [Revista de Pesquisa: Cuidado é Fundamental. Nursing & Research] [Revista de Enfermagem Atenção e Saùde] [Other journals in Portuguese]; [International Journal Of Nursing Studies] [Journal Of Nursing Scholarship] [European Journal Of Cardiovascular Nursing] [Nursing Outlook] [European Journal Of Cancer Care] [Birth-Issues In Perinatal Care] [Journal Of Advanced Nursing] [Worldviews On Evidence-Based Nursing] [Journal Of Cardiovascular Nursing] [Nurse Education Today] [American Journal Of Critical Care] [International Journal Of Mental Health Nursing] [Journal Of Family Nursing] [Australian Critical Care] [Journal Of Tissue Viability] [Journal Of Nursing Management] [Nursing Ethics] [Cancer Nursing] [Journal Of Human Lactation] [Women and Birth] [World Psychiatry][Diabetes Care][Stroke][American Journal of Clinical Nutrition][International Journal of Obesity][Resuscitation][International Journal of Behavioral Nutrition and Physical Activity][Nutrition Reviews] [Current Opinion in HIV and AIDS] [Advances in Nutrition] [Journal of the American Medical Directors Association][Journal of Pain and Symptom Management][International Journal of Nursing Studies][Journal of Palliative Medicine][Journal of Nursing Scholarship][Nurse Education Today][Nursing Outlook][Patient][Worldviews on Evidence-Based Nursing][Journal of Advanced Nursing] [other international journals]

9. What are the reasons why you choose to read in the selected journals? [It is available in the language I understand] [It is easy to read] [The articles help me to learn new aspects of my work/study] [The articles help me to apply them in my work/study] [The articles help me to broaden my skills] [I discuss with my colleagues aspects that I have read in the articles] [I use the articles to develop protocols and procedures/class work] [The journal is available at my institution] [I have an individual subscription] [It is open access] [It has peer review] [It has external accreditations] [It is indexed in scientific databases] [It is indexed in nursing databases] [It has an impact factor] [The publisher is prestigious] [The abstract is in other languages] [It is disseminated on social networks] [Other reasons].

   I understand the language

   I learn and apply what I have learnt

I develop protocols, procedures, work

I discuss with my colleagues

I have it available in my institution

I have an individual subscription

It is open access

It has peer review

It has an impact factor

It is indexed in scientific databases

It is indexed in nursing databases

The publisher has prestige

The abstract is in other languages

It is disseminated on social networks

10. What are your reasons for choosing to publish in the selected journals? It is available in the language I understand] [It is easy to read] [The articles published are in line with my work] [It is a nursing journal] [My colleagues read this journal] [To expand my curriculum] [I have it available in my institution] [I have an individual subscription] [It is open access] [It has peer review] [It has external accreditations] [It is indexed in scientific databases] [It is indexed in nursing databases] [It has an impact factor] [The publisher has prestige] [The abstract is in other languages] [It is disseminated on social networks] [Other reasons].
11. What are your reasons for choosing to reference the selected journals? It is available in the language I understand] [It is easy to read] [The articles published are in line with my work/study] [My colleagues publish in this journal] [I have it available in my institution] [I have an individual subscription] [It is open access] [It has peer review] [It has external accreditations] [It is indexed in scientific databases] [It is indexed in nursing databases] [It has an impact factor] [The publisher is prestigious] [The abstract is in other languages] [It is disseminated on social networks] [Other reasons].

You have finished the questionnaire, thank you very much for your collaboration. If you have any suggestions or comments, you can write them here.
