# Supplementary material for: Research, Reading, and Publication Habits of Nurses and Nursing Students Applied to Impact Journals: International Multicentre Study
Source: Int J Environ Res Public Health. 2023 Mar 7;20(6):4697. doi: 10.3390/ijerph20064697 (PMC10049027; doi:10.3390/ijerph20064697)
Supplement: Supplementary file 1 [file ijerph-20-04697-s001.zip › Table S2. 1Q_LcEE-CAPC Questionnaire factor analysis.pdf]

Table S2. 1Q_LcEE-CAPC Questionnaire factor analysis. Reasons for reading journals.

Rotated Component Matrix	Component												
	1	2	3	4	5	6	7	8	9	10	11	12	Variance %	Accumulated %
I understand the language Journals in Portuguese	0.73	0.114	0.157	-0.024	0.049	0.043	0.086	0.003	0.044	0.173	0.049	0.129		
I learn and apply what I have learnt Journals in Portuguese	0.707	0.051	0.108	0.176	0.138	0.134	-0.044	-0.017	0.189	0.021	0.042	0.01		
It is open access Journals in Portuguese	0.662	0.222	0.118	-0.026	-0.026	-0.015	0.187	0.322	0.011	0.061	-0.071	-0.137		
Develop protocols, procedures, work Journals in Portuguese	0.634	0.063	0.11	-0.001	0.441	0.128	0.029	0.011	-0.059	0.192	-0.091	-0.075	20.088	20.088
Indexed in nursing databases Journals in Spanish	0.161	0.765	0.055	0.058	0.091	0.052	-0.101	0.089	0.23	0.001	0.079	0.006		
Indexed in nursing databases Journals in English	0.045	0.709	0.185	0.256	0.041	0.063	0.082	0.049	0.065	0.064	0.014	0.067		
Indexed in scientific databases Journals in Spanish	0.12	0.703	0.193	0.022	0.202	0.082	-0.031	0.189	0.032	-0.067	0.072	-0.013		
Indexed in nursing databases Journals in Portuguese	0.553	0.626	0.018	0.06	-0.058	0.078	-0.042	0.097	-0.02	0.09	0.011	-0.032		
Indexed in scientific databases Journals in English	0.074	0.618	0.299	0.32	0.113	0.123	0.07	0.17	-0.105	0.032	-0.005	0.159		
Indexed in scientific databases Journals in Portuguese	0.535	0.568	0.252	0.055	0.017	0.077	0.065	0.062	-0.102	-0.008	0.032	0.074	6.88	26.968
It has peer review Journals in Spanish	0.017	0.159	0.783	0.08	0.072	0.058	-0.009	0.066	-0.029	-0.031	0.218	-0.123		
It has peer review Journals in Portuguese	0.328	0.195	0.687	-0.009	0.04	-0.095	0.041	0.059	-0.074	-0.098	0.086	0.15		
It has peer review Journals in English	0.13	0.131	0.676	0.045	0.087	0.044	0.04	0.095	-0.043	0.377	-0.058	0.129		
It has impact factor Journals in Portuguese	0.299	0.308	0.512	0.084	-0.11	0.032	-0.001	0.142	0.156	-0.209	-0.22	0.197		
It has impact factor Journals in Spanish	0.069	0.164	0.487	0.268	-0.063	0.24	-0.067	0.071	0.292	-0.104	-0.064	-0.156		
It has impact factor Journals in English	0.06	0.265	0.437	0.376	0.05	0.122	-0.015	0.283	0.101	0.005	-0.226	0.237	5.822	32.79

It is available at my institution Journals in English	0.002	0.178	0.115	0.768	-0.021	0.002	0.096	-0.112	-0.05	0.006	0.107	-0.043		
It is available at my institution Journals in Spanish	-0.01	0.108	0.055	0.687	0.033	0.046	0.009	0.028	0.11	0.085	0.08	-0.21		
I learn and apply what I have learnt Journals in English	0.17	0.05	0.01	0.541	0.378	0.129	0.109	0.188	0.182	-0.087	0.044	0.271		
I understand the language Journals in English	-0.008	0.077	0.218	0.526	0.353	0.059	0.078	0.236	0.006	0.003	0.026	0.285		
It is available at my institution Journals in Portuguese	0.434	0.122	-0.056	0.504	0.003	0.161	-0.054	0.156	-0.003	0.003	-0.096	-0.029	4.233	37.023
Develop protocols, procedures, work Journals in Spanish	0.036	0.136	0.054	-0.046	0.798	0.041	0.04	0.002	0.09	0.05	0.094	-0.131		
Develop protocols, procedures, work Journals in English	0.161	0.129	0.018	0.194	0.756	0.05	0.09	0.094	0.061	-0.015	-0.088	0.146	3.86	40.884
The abstract is in other languages Journals in English	-0.121	0.1	-0.019	0.062	0.051	0.721	0.07	0.214	0.056	0.095	-0.005	0.079		
The abstract is in other languages Journals in Spanish	0.261	0.13	0.081	0.057	-0.026	0.685	0.1	-0.076	0.051	-0.026	0.046	-0.124		
The abstract is in other languages Journals in Portuguese	0.177	0.035	0.057	0.074	0.107	0.66	0.017	0.098	-0.101	-0.084	0.108	0.059	3.665	44.549
It is disseminated on social networks Journals in English	0.001	0.061	-0.065	0.033	0.139	0.131	0.731	0.099	-0.017	0.082	-0.12	0.056		
It is disseminated on social networks Journals in Portuguese	0.21	-0.049	0.045	0.059	-0.012	-0.037	0.711	-0.055	0.034	-0.034	0.019	-0.03		
It is disseminated on social networks Journals in Spanish	-0.063	-0.059	0.012	0.037	-0.001	0.092	0.612	0.258	0.126	-0.038	0.348	0.013		
The publisher has prestige Journals in English	-0.132	0.332	0.226	0.072	0.178	0.282	0.361	0.004	0.069	0.333	-0.192	-0.136	3.41	47.959
It is open access Journals in Spanish	0.151	0.182	0.129	0.043	0.087	0.15	0.045	0.722	0.05	-0.144	0.043	-0.122		
It is open access Journals in English	0.105	0.28	0.149	0.099	0.018	0.076	0.239	0.632	-0.009	0.151	-0.042	-0.022		
Discuss with my colleagues Journals in Spanish	-0.075	0.079	-0.016	0.075	0.248	-0.037	0.033	0.067	0.727	0.057	0.228	-0.024	3.316	51.275
Discuss with my colleagues Journals in Portuguese	0.395	0.073	0.087	0.058	-0.031	-0.037	0.057	0.057	0.541	0.184	-0.032	0.318		
The publisher has prestige Journals in Portuguese	0.373	0.212	-0.045	-0.029	-0.092	0.042	0.076	0.064	0.424	-0.23	-0.31	-0.065		
The publisher has prestige Journals in Spanish	-0.155	0.257	0.223	0.095	0.193	0.191	0.226	-0.148	0.265	-0.19	0.143	-0.192	3.108	54.383

I have an individual subscription Journals in English	0.173	0.033	-0.035	0.025	-0.041	-0.056	-0.043	0.039	-0.025	0.779	0.124	-0.033		
Discuss with my colleagues Journals in English	0.238	-0.043	-0.009	0.036	0.101	0.059	0.162	-0.085	0.465	0.584	-0.062	0.007	2.941	57.324
I have an individual subscription Journals in Spanish	-0.017	0.106	0.05	0.046	-0.047	0.069	0.042	-0.035	0.026	0.053	0.667	0.015		
I understand the language Journals in Spanish	0.005	0.062	0.106	0.089	0.256	0.111	-0.082	0.46	0.148	0.118	0.463	0.078		
I learn and apply what I have learnt Journals in Spanish	0.066	-0.027	-0.061	0.268	0.421	0.038	0.039	0.171	0.303	-0.165	0.431	-0.043	2.799	60.123
I have an individual subscription Journals in Portuguese	-0.013	0.083	0.067	-0.077	-0.006	0.01	-0.001	-0.104	0.023	-0.03	0.038	0.788	2.509	62.633
Extraction method: Main component analysis. Rotation method: Varimax with Kaiser normalisation.														
Rotation has converged in 8 iterations.														
KMO and Bartlett's test														
Kaiser-Meyer-Olkin measure of sampling adequacy		0.834												
Bartlett's test of sphericity Approximate Chi-squared		6187.284												
	gl	861												
	Sig.	0												
Total explained variance														
Component	Initial eigenvalues			Sum of the squared saturations of the extraction			Sum of the squared saturations of the rotation							
	Total	Variance %	Accumulated %	Total	Variance %	Accumulated %	Total	Variance %	Accumulated %					
1	8.437	20.088	20.088	8.437	20.088	20.088	3.608	8.591	8.591					
2	2.89	6.88	26.968	2.89	6.88	26.968	3.529	8.403	16.994					
3	2.445	5.822	32.79	2.445	5.822	32.79	2.782	6.624	23.618					

4	1.778	4.233	37.023	1.778	4.233	37.023	2.507	5.97	29.588					
5	1.621	3.86	40.884	1.621	3.86	40.884	2.232	5.315	34.902					
6	1.539	3.665	44.549	1.539	3.665	44.549	1.842	4.385	39.288					
7	1.432	3.41	47.959	1.432	3.41	47.959	1.841	4.384	43.671					
8	1.393	3.316	51.275	1.393	3.316	51.275	1.809	4.308	47.979					
9	1.305	3.108	54.383	1.305	3.108	54.383	1.752	4.172	52.151					
10	1.235	2.941	57.324	1.235	2.941	57.324	1.612	3.837	55.988					
11	1.176	2.799	60.123	1.176	2.799	60.123	1.463	3.484	59.472					
12	1.054	2.509	62.633	1.054	2.509	62.633	1.327	3.16	62.633					
13	0.988	2.352	64.985											
14	0.958	2.282	67.266											
15	0.888	2.114	69.38											
16	0.878	2.091	71.472											
17	0.815	1.94	73.411											
18	0.777	1.849	75.26											
19	0.719	1.712	76.972											
20	0.672	1.601	78.573											
21	0.658	1.566	80.139											
22	0.609	1.45	81.589											
23	0.579	1.378	82.967											

24	0.562	1.337	84.304											
25	0.538	1.28	85.585											
26	0.521	1.24	86.825											
27	0.497	1.183	88.008											
28	0.459	1.093	89.102											
29	0.444	1.056	90.158											
30	0.425	1.012	91.17											
31	0.413	0.982	92.152											
32	0.395	0.941	93.093											
33	0.371	0.883	93.976											
34	0.364	0.866	94.843											
35	0.352	0.838	95.681											
36	0.332	0.79	96.471											
37	0.315	0.751	97.223											
38	0.274	0.653	97.876											
39	0.253	0.604	98.479											
40	0.242	0.576	99.055											
41	0.208	0.495	99.549											
42	0.189	0.451	100											
