# Supplementary material for: Research, Reading, and Publication Habits of Nurses and Nursing Students Applied to Impact Journals: International Multicentre Study
Source: Int J Environ Res Public Health. 2023 Mar 7;20(6):4697. doi: 10.3390/ijerph20064697 (PMC10049027; doi:10.3390/ijerph20064697)
Supplement: Supplementary file 1 [file ijerph-20-04697-s001.zip › Table S3. 1Q_LcEE-CAPC Questionnaire. Hypothesis, demographic and responses.pdf]

Table S3. 1Q_LcEE-CAPC Questionnaire. Contrast of hypothesis, demographic variables, and responses to reading, referencing, publishing, knowing, and not knowing journals in Spanish, Portuguese, and English.

1. Contrast of hypothesis, demographic variables, and responses to ‘reading journals in Spanish, Portuguese, and English’.

Order	Journals	Total	Percentage	Language*	Country*	Sex*	Experience* (years/percentile)	Age* (years)	Situation* working/studying	Public work* Private work	Primary care* Hospital care	Job profile*
1	Index de Enfermería*	180	45.7% 180	0.000	0.000	0.072	0.013	0.004	0.000	0.030	0.461	0.011
2	Investigación y Educación en Enfermería	126	32% 126	0.479	0.484	0.144	0.223	0.774	0.077	0.165	0.631	0.033
3	Aquichán	35	8.9% 35	0.365	0.450	0.205	0.012	0.026	0.001	0.040	0.398	0.000
4	Enfermería Intensiva	92	23.4% 92	0.021	0.081	0.001	0.309	0.292	0.050	0.079	0.293	0.012
5	Enfermería universitaria	57	14.5% 57	0.731	0.813	0.673	0.052	0.327	0.880	0.248	0.143	0.321
6	Enfermería Global	117	29.7% 117	0.047	0.190	0.890	0.305	0.070	0.017	0.007	0.330	0.000
7	Enfermería Nefrológica	66	16.8% 66	0.000	0.007	0.266	0.110	0.078	0.644	0.209	0.627	0.353
8	Revista ENE de Enfermería	100	25.4% 100	0.000	0.000	0.047	0.001	0.000	0.000	0.017	0.010	0.000
9	Revista CUIDARTE	99	25.1% 99	0.001	0.020	0.494	0.019	0.068	0.660	0.488	0.306	0.258
10	Avances en Enfermería	74	18.8% 74	0.160	0.487	0.776	0.197	0.052	0.038	0.650	0.577	0.349
11	Enfermería Clínica	148	37.6% 148	0.000	0.000	0.003	0.000	0.000	0.000	0.016	0.095	0.003

12	Cultura de los Cuidados	87	22.1%<br>87	0.000	0.000	0.027	0.000	0.000	0.000	0.129	0.208	0.012
13	Investigación en enfermería: imagen y desarrollo	38	9.6%<br>38	0.006	0.110	0.727	0.447	0.152	0.467	0.614	0.774	0.993
14	Temperamentvm	29	7.4%<br>29	0.002	0.006	0.087	0.000	0.000	0.001	0.004	0.093	0.013
15	Gerokomos	64	16.2%<br>64	0.000	0.000	0.001	0.000	0.000	0.000	0.004	0.008	0.000
16	Revista Ética de los Cuidados	60	15.2%<br>60	0.000	0.000	0.579	0.000	0.000	0.000	0.115	0.507	0.154
17	Archivos de la Memoria	30	7.6%<br>30	0.006	0.016	0.680	0.072	0.025	0.870	0.033	0.436	0.906
18	Revista Tesela	30	7.6%<br>30	0.002	0.005	0.023	0.000	0.000	0.009	0.543	0.654	0.173
19	Revista Cubana de Enfermería	84	21.3%<br>84	0.001	0.013	0.671	0.357	0.479	0.037	0.072	0.819	0.148
20	Revista de Enfermería del Instituto Mexicano del Seguro Social	23	5.8%<br>23	0.000	0.000	0.616	0.099	0.226	0.292	0.595	0.249	0.973
21	Metas de Enfermería	122	31.0%<br>122	0.000	0.000	0.005	0.000	0.000	0.000	0.002	0.000	0.000
22	Enfermería Comunitaria. Revista internacional de cuidados de salud familiar y comunitaria	117	29.7%<br>117	0.000	0.000	0.014	0.000	0.000	0.000	0.074	0.000	0.000
23	Matronas Profesión	28	7.1%<br>28	0.001	0.039	0.893	0.007	0.047	0.430	0.605	0.885	0.831
24	Revista Rol de Enfermería	135	34.3%<br>135	0.000	0.000	0.000	0.000	0.000	0.000	0.015	0.000	0.000
25	Enfermería Docente	48	12.2%<br>48	0.001	0.021	0.215	0.007	0.026	0.016	0.637	0.660	0.064
26	Biblioteca Lascasas	41	10.4%<br>41	0.001	0.005	0.301	0.000	0.000	0.000	0.050	0.122	0.014

27	Revista da Escola de Enfermagem da USP	81	20.6%<br>81	0.000	0.000	0.824	0.982	0.810	0.411	0.307	0.050	0.000
28	Texto & Contexto: Enfermagem	78	19.8%<br>78	0.000	0.000	0.763	0.671	0.808	0.361	0.209	0.119	0.023
29	Escola Anna Nery Revista de Enfermagem	50	12.7%<br>50	0.000	0.000	0.485	0.305	0.128	0.000	0.412	0.019	0.043
30	Revista Latino-Americana de Enfermagem	93	23.6%<br>93	0.000	0.000	0.921	0.830	0.917	0.735	0.521	0.020	0.002
31	Brasileira de Enfermagem	82	20.8%<br>82	0.000	0.000	0.351	0.625	0.738	0.003	0.462	0.095	0.041
32	Revista Gaúcha de Enfermagem	55	14.0%<br>55	0.000	0.000	0.404	0.394	0.375	0.158	0.407	0.111	0.016
33	ACTA Paulista de Enfermagem	63	16.0%<br>63	0.000	0.000	0.719	0.997	0.539	0.015	0.320	0.014	0.006
34	Ciência, Cuidado e Saúde	64	16.2%<br>64	0.000	0.000	0.499	0.951	0.680	0.168	0.678	0.167	0.042
35	Revista Eletrônica de Enfermagem	71	18.0%<br>71	0.000	0.000	0.324	0.925	0.296	0.001	0.430	0.125	0.021
36	Revista da Rede de Enfermagem do Nordeste	42	10.7%<br>42	0.000	0.000	0.733	0.099	0.074	0.012	0.338	0.045	0.015
37	Cogitare Enfermagem	41	10.4%<br>41	0.000	0.000	0.350	0.770	0.721	0.099	0.508	0.086	0.006
38	Revista de Enfermagem da UFSM	43	10.9%<br>43	0.000	0.000	0.712	0.390	0.164	0.064	0.260	0.045	0.066
39	Revista Mineira de Enfermagem	43	10.9%<br>43	0.000	0.000	0.804	0.115	0.144	0.017	0.660	0.139	0.040
40	Revista Enfermagem em Foco	45	11.4%<br>45	0.000	0.000	0.784	0.212	0.104	0.002	0.722	0.046	0.018
41	Revista SOBECC	26	6.6%<br>26	0.000	0.000	0.814	0.155	0.021	0.004	0.438	0.173	0.715
42	Revista de Enfermagem Referência	31	7.9%<br>31	0.000	0.000	0.880	0.893	0.995	0.119	0.513	0.133	0.121

43	Revista de Enfermagem do Centro-Oeste Mineiro	14	3.6%<br>14	0.000	0.000	0.915	0.385	0.114	0.177	0.462	0.022	0.993
44	Revista de Enfermagem UFPE On Line	37	9.4%<br>37	0.000	0.000	0.842	0.030	0.034	0.016	0.408	0.044	0.026
45	Revista Baiana de Enfermagem	36	9.1%<br>36	0.000	0.000	0.354	0.391	0.297	0.199	0.480	0.267	0.023
46	Revista de Enfermagem da Universidade Federal do Piauí	23	5.8%<br>23	0.000	0.000	0.324	0.178	0.288	0.018	0.282	0.075	0.210
47	Revista de Pesquisa: Cuidado é Fundamental	35	8.9%<br>35	0.000	0.000	0.307	0.665	0.520	0.910	0.007	0.341	0.104
48	Revista de Enfermagem e Atenção a Saùde	30	7.6%<br>30	0.000	0.001	0.831	0.615	0.568	0.241	0.735	0.205	0.432
49	International Journal of Nursing Studies	128	32.5%<br>128	0.028	0.107	0.247	0.026	0.602	0.125	0.060	0.646	0.036
50	Journal of Nursing Scholarship	60	15.2%<br>60	0.534	0.925	0.088	0.280	0.657	0.009	0.268	0.540	0.003
51	European Journal of Cardiovascular Nursing	53	13.5%<br>53	0.375	0.285	0.631	0.861	0.588	0.641	0.147	0.881	0.984
52	Nursing Outlook	46	11.7%<br>46	0.037	0.298	0.017	0.008	0.061	0.001	0.078	0.866	0.027
53	European Journal of Cancer Care	39	9.9%<br>39	0.090	0.541	0.790	0.503	0.514	0.250	0.341	0.872	0.033
54	Birth-issues in perinatal care	12	3.0%<br>12	0.625	0.978	0.575	0.846	0.302	0.360	0.644	0.981	0.997
55	Journal of advanced Nursing	92	23.4%<br>92	0.027	0.106	0.517	0.016	0.040	0.001	0.019	0.547	0.000
56	Worldviews on Evidence-Based Nursing	26	6.6%<br>26	0.009	0.001	0.154	0.093	0.062	0.007	0.056	0.905	0.600
57	Journal of Cardiovascular Nursing	43	10.9%<br>43	0.072	0.053	0.423	0.504	0.679	0.085	0.694	0.422	0.841
58	Nurse Education Today	58	14.7%<br>58	0.050	0.178	0.182	0.016	0.016	0.001	0.061	0.823	0.000

59	American Journal of Critical Care	64	16.2%<br>64	0.459	0.408	0.140	0.279	0.771	0.132	0.052	0.842	0.117
60	International Journal of Mental Health Nursing	59	15.0%<br>59	0.396	0.340	0.739	0.519	0.166	0.614	0.468	0.558	0.642
61	Journal of Family Nursing	40	10.2%40	0.325	0.869	0.740	0.602	0.300	0.028	0.014	0.123	0.053
62	Australian Critical Care	17	4.3%<br>17	0.597	0.981	0.324	0.257	0.678	0.453	0.402	0.810	0.046
63	Journal of Tissue Viability	11	2.8%<br>11	0.390	0.927	0.051	0.226	0.777	0.943	0.743	0.619	0.005
64	Journal of Nursing Management	43	10.9%<br>43	0.801	0.983	0.373	0.005	0.022	0.032	0.192	0.753	0.001
65	Nursing Ethics	33	8.4%<br>33	0.061	0.022	0.708	0.000	0.003	0.001	0.123	0.239	0.000
66	Cancer Nursing	22	5.6%<br>22	0.694	0.946	0.919	0.247	0.395	0.476	0.729	0.824	0.439
67	Journal of Human Lactation	8	2.0%<br>8	0.696	0.985	0.957	0.354	0.823	0.576	0.324	0.556	0.944
68	Women and Birth	16	4.1%<br>16	0.391	0.911	0.602	0.353	0.477	0.340	0.597	0.571	0.808
69	World Psychiatry	20	5.1%<br>20	0.522	0.006	0.017	0.178	0.145	0.946	0.673	0.084	0.446
70	Diabetes Care	70	17.8%<br>70	0.408	0.870	0.075	0.163	0.402	0.528	0.883	0.908	0.165
71	Stroke	15	3.8%<br>15	0.356	0.914	0.315	0.780	0.719	0.783	0.910	0.060	0.398
72	American Journal of Clinical Nutrition	36	9.1%<br>36	0.840	0.994	0.349	0.601	0.242	0.991	0.278	0.409	0.190
73	International Journal of Obesity	34	8.6%<br>34	0.173	0.731	0.866	0.271	0.261	0.810	0.956	0.002	0.559
74	Resucitation	13	8.4%<br>13	0.986	0.152	0.157	0.882	0.980	0.316	0.688	0.120	0.028

75	International Journal of Behavioral Nutrition and Physical Activity	20	5.1%<br>20	0.675	0.291	0.919	0.616	0.314	0.476	0.309	0.925	0.146
76	Nutrition Reviews	18	4.6%<br>18	0.823	0.222	0.923	0.366	0.268	0.463	0.239	0.093	0.661
77	Current Opinion in HIV and AIDS	11	2.8%<br>11	0.876	0.020	0.936	0.087	0.548	0.585	0.682	0.503	0.414
78	Advances in Nutrition	21	5.3%<br>21	0.733	0.955	0.142	0.849	0.864	0.802	0.842	0.157	0.721
79	Journal of the American Medical Directors Association	13	3.3%<br>13	0.016	0.005	0.639	0.787	0.308	0.786	0.580	0.981	0.216
80	Journal of Pain and Symptom Management	24	6.1%<br>24	0.682	0.975	0.099	0.271	0.369	0.061	0.006	0.179	0.661
81	Journal of Palliative Medicine	38	9.6%<br>38	0.602	0.963	0.731	0.412	0.045	0.712	0.467	0.335	0.328
82	Patient	14	3.6%<br>14	0.247	0.810	0.825	0.277	0.978	0.909	0.917	0.470	0.934

2. Contrast of hypotheses, demographic variables, and responses to ‘referencing journals in Spanish, Portuguese, and English’.

Order	Jjournals	Total	Percentage Total	Language*	Country*	Sex*	Experience* (years)	Age* (years)	Situation* working/studying	Public work* Private work	Primary care* Hospital care	Job profile*
1	Index de Enfermería*	83	21.1% 83	0.000	0.001	0.045	0.038	0.038	0.037	0.187	0.954	0.000
2	Investigación y Educación en Enfermería	41	10.4% 41	0.088	0.496	0.206	0.454	0.183	0.024	0.307	0.002	0.000
3	Aquichán	24	6.1% 24	0.420	0.317	0.907	0.274	0.129	0.004	0.031	0.104	0.000
4	Enfermería Intensiva	30	7.6% 30	0.560	0.407	0.118	0.082	0.151	0.189	0.495	0.017	0.000
5	Enfermería universitaria	15	3.8% 15	0.136	0.605	0.139	0.033	0.232	59.000	0.794	0.359	0.865
6	Enfermería Global	70	17.8% 70	0.259	0.527	0.628	0.795	0.528	0.274	0.260	0.045	0.000
7	Enfermería Nefrológica	23	5.8% 23	0.001	0.045	0.871	0.383	0.272	0.160	0.012	0.626	0.995
8	Revista ENE de Enfermería	43	10.9% 43	0.000	0.003	0.496	0.239	0.772	0.976	0.671	0.505	0.126
9	Revista CUIDARTE	30	7.6% 30	0.662	0.976	0.022	0.511	0.631	0.279	0.624	0.592	0.933
10	Avances en Enfermería	26	6.6% 26	0.679	0.440	0.494	0.194	0.693	0.756	0.254	0.123	0.004
11	Enfermería Clínica	77	19.5% 77	0.000	0.002	0.351	0.002	0.017	0.008	0.009	0.191	0.000
12	Cultura de los Cuidados	34	8.6% 34	0.160	0.054	0.566	0.272	0.204	0.009	0.101	0.381	0.009

13	Investigación en enfermería: imagen y desarrollo	14	3.6%<br>14	0.482	0.907	0.406	0.732	0.256	0.208	0.566	0.020	0.228
14	Temperamentvm	13	3.3%<br>13	0.118	0.014	0.639	0.045	0.074	0.145	0.324	0.635	0.956
15	Gerokomos	33	8.4%<br>33	0.000	0.009	0.121	0.000	0.006	0.000	0.033	0.060	0.000
16	Revista Ética de los Cuidados	13	3.3%<br>13	0.398	0.930	0.889	0.023	0.042	0.145	0.128	0.012	0.027
17	Archivos de la Memoria	8	2.0%<br>8	0.437	0.946	0.079	0.239	0.450	0.847	0.335	0.598	0.972
18	Revista Tesela	8	2.0%<br>8	0.437	0.946	0.896	0.908	0.820	0.951	0.200	0.000	0.003
19	Revista Cubana de Enfermería	84	21.3%<br>84	0.001	0.364	0.185	0.405	0.875	0.804	0.405	0.325	0.003
20	Revista de Enfermería del Instituto Mexicano del Seguro Social	23	5.8%<br>23	0.000	0.011	0.678	0.223	0.085	0.059	0.289	0.008	0.801
21	Metas de Enfermería	122	31.0%<br>122	0.000	0.000	0.368	0.001	0.000	0.000	0.013	0.246	0.000
22	Enfermería Comunitaria. Revista internacional de cuidados de salud familiar y comunitaria	117	29.7%<br>117	0.000	0.088	0.463	0.276	0.296	0.193	0.026	0.562	0.848
23	Matronas Profesión	28	7.1%<br>28	0.001	0.650	0.863	0.548	0.807	0.491	0.234	0.000	0.005
24	Revista Rol de Enfermería	135	34.3%<br>135	0.000	0.001	0.233	0.013	0.003	0.007	0.263	0.043	0.001
25	Enfermería Docente	48	12.2%<br>48	0.001	0.512	0.152	0.114	0.818	0.646	0.354	0.000	0.000
26	Biblioteca Lascasas	41	10.4%<br>41	0.001	0.752	0.517	0.314	0.886	0.235	0.569	0.037	0.512
27	Revista da Escola de Enfermagem da USP	52	13.2%<br>52	0.000	0.000	0.784	0.682	0.616	0.870	0.146	0.260	0.000

28	Texto & Contexto: Enfermagem	52	13.2%<br>52	0.000	0.000	0.250	0.919	0.338	0.892	0.341	0.006	0.000
29	Escola Anna Nery Revista de Enfermagem	323	8.1%<br>32	0.000	0.000	0.810	0.094	0.218	0.005	0.680	0.177	0.000
30	Revista Latino-Americana de Enfermagem	55	14.0%<br>55	0.000	0.000	0.746	0.242	0.065	0.233	0.259	0.168	0.006
31	Brasileira de Enfermagem	55	14.0%<br>55	0.000	0.000	0.378	0.168	0.117	0.003	0.489	0.002	0.001
32	Revista Gaúcha de Enfermagem	41	10.4%<br>41	0.000	0.000	0.529	0.038	0.040	0.040	0.679	0.038	0.000
33	ACTA Paulista de Enfermagem	40	10.2%<br>40	0.000	0.000	0.244	0.320	0.171	0.029	0.662	0.017	0.016
34	Ciência, Cuidado e Saúde	31	7.9%<br>31	0.000	0.000	0.650	0.485	0.424	0.330	0.766	0.256	0.015
35	Revista Eletrônica de Enfermagem	40	10.2%<br>40	0.000	0.000	0.623	0.215	0.044	0.020	0.622	0.113	0.023
36	Revista da Rede de Enfermagem do Nordeste	25	6.3%<br>25	0.000	0.000	0.437	0.258	0.303	0.870	0.195	0.358	0.001
37	Cogitare Enfermagem	26	6.6%<br>26	0.000	0.000	0.336	0.046	0.009	0.007	0.581	0.231	0.007
38	Revista de Enfermagem da UFSM	24	6.1%<br>24	0.000	0.000	0.729	0.168	0.027	0.756	0.149	0.026	0.052
39	Revista Mineira de Enfermagem	20	5.1%<br>20	0.000	0.000	0.798	0.063	0.022	0.292	0.814	0.067	0.009
40	Revista Enfermagem em Foco	18	4.6%<br>18	0.000	0.000	0.903	0.067	0.001	0.000	0.253	0.212	0.509
41	Revista SOBECC	5	1.3%<br>5	0.000	0.476	0.709	0.580	0.006	0.037	0.048	0.000	0.008
42	Revista de Enfermagem Referência	19	4.8%<br>19	0.000	0.000	0.343	0.155	0.137	0.024	0.155	0.132	0.336
43	Revista de Enfermagem do Centro-Oeste Mineiro	8	2.0%<br>8	0.001	0.024	0.433	0.349	0.092	0.290	0.765	0.410	0.962

44	Revista de Enfermagem UFPE On Line	29	7.4%<br>29	0.000	0.000	0.793	0.067	0.035	0.159	0.330	0.152	0.018
45	Revista Baiana de Enfermagem	18	4.6%<br>18	0.000	0.000	0.547	0.060	0.017	0.257	0.753	0.658	0.015
46	Revista de Enfermagem da Universidade Federal do Piauí	10	2.5%<br>10	0.000	0.001	0.936	0.330	0.231	0.245	0.682	0.106	0.304
47	Revista de Pesquisa: Cuidado é Fundamental	24	6.1%<br>24	0.000	0.000	0.616	0.109	0.080	0.120	0.039	0.192	0.569
48	Revista de Enfermagem e Atenção a Saùde	12	3.0%<br>12	0.043	0.394	0.425	0.253	0.146	0.304	0.832	0.331	0.955
49	International Journal of Nursing Studies	64	16.2%<br>64	0.031	0.305	0.565	0.089	0.428	0.995	0.060	0.573	0.139
50	Journal of Nursing Scholarship	38	9.6%<br>38	0.044	0.382	0.852	0.181	0.544	0.057	0.159	0.267	0.000
51	European Journal of Cardiovascular Nursing	26	6.6%<br>26	0.852	0.994	0.729	0.017	0.024	0.047	0.031	0.188	0.752
52	Nursing Outlook	17	4.3%<br>17	0.034	0.339	0.606	0.205	0.594	0.213	0.245	0.070	0.001
53	European Journal of Cancer Care	19	4.8%<br>19	0.591	0.953	0.894	0.198	0.063	0.001	0.114	0.150	0.091
54	Birth-issues in perinatal care	8	2.0%<br>8	0.165	0.582	0.165	0.259	0.350	0.517	0.256	0.481	0.887
55	Journal of advanced Nursing	57	14.5%<br>57	0.081	0.490	0.641	0.084	0.067	0.003	0.008	0.461	0.002
56	Worldviews on Evidence-Based Nursing	17	4.3%<br>17	0.353	0.848	0.799	0.789	0.611	0.270	0.467	0.042	0.025
57	Journal of Cardiovascular Nursing	29	7.4%<br>29	0.913	0.990	0.455	0.205	0.561	0.279	0.323	0.095	0.687
58	Nurse Education Today	32	8.1%<br>32	0.056	0.437	0.793	0.199	0.228	0.042	0.164	0.045	0.000
59	American Journal of Critical Care	27	6.9%<br>27	0.392	0.915	0.319	0.703	0.223	0.026	0.595	0.220	0.391

60	International Journal of Mental Health Nursing	26	6.6%<br>26	0.531	0.947	0.843	0.579	0.143	0.374	0.230	0.029	0.024
61	Journal of Family Nursing	24	6.1%24	0.741	0.992	0.873	0.558	0.203	0.323	0.449	0.104	0.000
62	Australian Critical Care	13	3.3%<br>13	0.656	0.983	0.639	0.939	0.749	0.786	0.410	0.023	0.001
63	Journal of Tissue Viability	8	2.0%<br>8	0.696	0.985	0.079	0.486	0.726	0.847	0.031	0.824	0.042
64	Journal of Nursing Management	28	7.1%<br>28	0.783	0.995	0.813	0.217	0.326	0.232	0.401	0.112	0.006
65	Nursing Ethics	13	3.3%<br>13	0.269	0.830	0.206	0.307	0.294	0.302	0.338	0.038	0.001
66	Cancer Nursing	10	2.5%<br>10	0.539	0.960	0.433	0.115	0.028	0.016	0.408	0.002	0.065
67	Journal of Human Lactation	7	1.8%<br>7	0.147	0.531	0.248	0.689	0.329	0.630	0.783	0.318	0.721
68	Women and Birth	11	2.8%<br>11	0.066	0.501	0.301	0.898	0.392	0.943	0.954	0.005	0.273
69	World Psychiatry	8	2.0%<br>8	0.575	0.979	0.794	0.542	0.230	0.576	0.224	0.960	0.267
70	Diabetes Care	27	6.9%<br>27	0.392	0.915	0.179	0.202	0.872	0.870	0.556	0.301	0.178
71	Stroke	8	2.0%<br>8	0.696	0.985	0.896	0.144	0.270	0.951	0.731	0.000	0.000
72	American Journal of Clinical Nutrition	22	5.6%<br>22	0.878	0.981	0.405	0.763	0.656	0.688	0.667	0.865	0.257
73	International Journal of Obesity	17	4.3%<br>17	0.202	0.717	0.799	0.154	0.469	0.930	0.441	0.772	0.532
74	Resucitation	10	2.5%<br>10	0.214	0.770	0.387	0.933	0.570	0.883	0.626	0.003	0.000
75	International Journal of Behavioral Nutrition and Physical Activity	10	2.5%<br>10	0.632	0.979	0.745	0.127	0.210	0.619	0.665	0.008	0.665

76	Nutrition Reviews	11	2.8%<br>11	0.923	0.999	0.301	0.404	0.536	0.585	0.525	0.819	0.525
77	Current Opinion in HIV and AIDS	6	1.5%<br>6	0.850	0.999	0.930	0.929	0.830	0.960	0.081	0.000	0.081
78	Advances in Nutrition	11	2.8%<br>11	0.733	0.995	0.516	0.869	0.259	0.491	0.224	0.012	0.224
79	Journal of the American Medical Directors Association	5	1.3%<br>5	0.553	0.952	0.912	0.707	0.836	0.787	0.423	0.000	0.423
80	Journal of Pain and Symptom Management	13	3.3%<br>13	0.638	0.981	0.249	0.509	0.995	0.786	0.856	0.015	0.856
81	Journal of Palliative Medicine	19	4.8%<br>19	0.339	0.884	0.750	0.403	0.138	0.799	0.514	0.071	0.514
82	Patient	4	1.0%<br>4	0.464	0.956	0.979	0.131	0.629	0.085	0.595	0.863	0.676

3. Contrast of hypotheses, demographic variables, and responses to ‘publishing in journals in Spanish, Portuguese, and English’.

Order	Journals	Total	Percentage Total	Language*	Country*	Sex*	Experience* (years)	Age* (years)	Situation* working/studying	Public work* Private work	Primary care* Hospital care	Job profile*
1	Index de Enfermería*	83	21.1% 83	0.000	0.001	0.045	0.038	0.038	0.037	0.187	0.954	0.000
2	Investigación y Educación en Enfermería	41	10.4% 41	0.088	0.496	0.206	0.454	0.183	0.024	0.307	0.002	0.000
3	Aquichán	24	6.1% 24	0.420	0.317	0.907	0.274	0.129	0.004	0.031	0.104	0.000
4	Enfermería Intensiva	30	7.6% 30	0.560	0.407	0.118	0.082	0.151	0.189	0.495	0.017	0.000
5	Enfermería universitaria	15	3.8% 15	0.136	0.605	0.139	0.033	0.232	59.000	0.794	0.359	0.865
6	Enfermería Global	70	17.8% 70	0.259	0.527	0.628	0.795	0.528	0.274	0.260	0.045	0.000
7	Enfermería Nefrológica	23	5.8% 23	0.001	0.045	0.871	0.383	0.272	0.160	0.012	0.626	0.995
8	Revista ENE de Enfermería	43	10.9% 43	0.000	0.003	0.496	0.239	0.772	0.976	0.671	0.505	0.126
9	Revista CUIDARTE	30	7.6% 30	0.662	0.976	0.022	0.511	0.631	0.279	0.624	0.592	0.933
10	Avances en Enfermería	26	6.6% 26	0.679	0.440	0.494	0.194	0.693	0.756	0.254	0.123	0.004
11	Enfermería Clínica	77	19.5% 77	0.000	0.002	0.351	0.002	0.017	0.008	0.009	0.191	0.000

12	Cultura de los Cuidados	34	8.6%<br>34	0.160	0.054	0.566	0.272	0.204	0.009	0.101	0.381	0.009
13	Investigación en enfermería: imagen y desarrollo	14	3.6%<br>14	0.482	0.907	0.406	0.732	0.256	0.208	0.566	0.020	0.228
14	Temperamentvm	13	3.3%<br>13	0.118	0.014	0.639	0.045	0.074	0.145	0.324	0.635	0.956
15	Gerokomos	33	8.4%<br>33	0.000	0.009	0.121	0.000	0.006	0.000	0.033	0.060	0.000
16	Revista Ética de los Cuidados	13	3.3%<br>13	0.398	0.930	0.889	0.023	0.042	0.145	0.128	0.012	0.027
17	Archivos de la Memoria	8	2.0%<br>8	0.437	0.946	0.079	0.239	0.450	0.847	0.335	0.598	0.972
18	Revista Tesela	8	2.0%<br>8	0.437	0.946	0.896	0.908	0.820	0.951	0.200	0.000	0.003
19	Revista Cubana de Enfermería	84	21.3%<br>84	0.001	0.364	0.185	0.405	0.875	0.804	0.405	0.325	0.003
20	Revista de Enfermería del Instituto Mexicano del Seguro Social	23	5.8%<br>23	0.000	0.011	0.678	0.223	0.085	0.059	0.289	0.008	0.801
21	Metas de Enfermería	122	31.0%<br>122	0.000	0.000	0.368	0.001	0.000	0.000	0.013	0.246	0.000
22	Enfermería Comunitaria. Revista internacional de cuidados de salud familiar y comunitaria	117	29.7%<br>117	0.000	0.088	0.463	0.276	0.296	0.193	0.026	0.562	0.848
23	Matronas Profesión	28	7.1%<br>28	0.001	0.650	0.863	0.548	0.807	0.491	0.234	0.000	0.005
24	Revista Rol de Enfermería	135	34.3%<br>135	0.000	0.001	0.233	0.013	0.003	0.007	0.263	0.043	0.001
25	Enfermería Docente	48	12.2%<br>48	0.001	0.512	0.152	0.114	0.818	0.646	0.354	0.000	0.000
26	Biblioteca Lascasas	41	10.4%<br>41	0.001	0.752	0.517	0.314	0.886	0.235	0.569	0.037	0.512

27	Revista da Escola de Enfermagem da USP	52	13.2%<br>52	0.000	0.000	0.784	0.682	0.616	0.870	0.146	0.260	0.000
28	Texto & Contexto: Enfermagem	52	13.2%<br>52	0.000	0.000	0.250	0.919	0.338	0.892	0.341	0.006	0.000
29	Escola Anna Nery Revista de Enfermagem	323	8.1%<br>32	0.000	0.000	0.810	0.094	0.218	0.005	0.680	0.177	0.000
30	Revista Latino-Americana de Enfermagem	55	14.0%<br>55	0.000	0.000	0.746	0.242	0.065	0.233	0.259	0.168	0.006
31	Brasileira de Enfermagem	55	14.0%<br>55	0.000	0.000	0.378	0.168	0.117	0.003	0.489	0.002	0.001
32	Revista Gaúcha de Enfermagem	41	10.4%<br>41	0.000	0.000	0.529	0.038	0.040	0.040	0.679	0.038	0.000
33	ACTA Paulista de Enfermagem	40	10.2%<br>40	0.000	0.000	0.244	0.320	0.171	0.029	0.662	0.017	0.016
34	Ciência, Cuidado e Saúde	31	7.9%<br>31	0.000	0.000	0.650	0.485	0.424	0.330	0.766	0.256	0.015
35	Revista Eletrônica de Enfermagem	40	10.2%<br>40	0.000	0.000	0.623	0.215	0.044	0.020	0.622	0.113	0.023
36	Revista da Rede de Enfermagem do Nordeste	25	6.3%<br>25	0.000	0.000	0.437	0.258	0.303	0.870	0.195	0.358	0.001
37	Cogitare Enfermagem	26	6.6%<br>26	0.000	0.000	0.336	0.046	0.009	0.007	0.581	0.231	0.007
38	Revista de Enfermagem da UFSM	24	6.1%<br>24	0.000	0.000	0.729	0.168	0.027	0.756	0.149	0.026	0.052
39	Revista Mineira de Enfermagem	20	5.1%<br>20	0.000	0.000	0.798	0.063	0.022	0.292	0.814	0.067	0.009
40	Revista Enfermagem em Foco	18	4.6%<br>18	0.000	0.000	0.903	0.067	0.001	0.000	0.253	0.212	0.509
41	Revista SOBECC	5	1.3%<br>5	0.000	0.476	0.709	0.580	0.006	0.037	0.048	0.000	0.008
42	Revista de Enfermagem Referência	19	4.8%<br>19	0.000	0.000	0.343	0.155	0.137	0.024	0.155	0.132	0.336

43	Revista de Enfermagem do Centro-Oeste Mineiro	8	2.0%<br>8	0.001	0.024	0.433	0.349	0.092	0.290	0.765	0.410	0.962
44	Revista de Enfermagem UFPE On Line	29	7.4%<br>29	0.000	0.000	0.793	0.067	0.035	0.159	0.330	0.152	0.018
45	Revista Baiana de Enfermagem	18	4.6%<br>18	0.000	0.000	0.547	0.060	0.017	0.257	0.753	0.658	0.015
46	Revista de Enfermagem da Universidade Federal do Piauí	10	2.5%<br>10	0.000	0.001	0.936	0.330	0.231	0.245	0.682	0.106	0.304
47	Revista de Pesquisa: Cuidado é Fundamental	24	6.1%<br>24	0.000	0.000	0.616	0.109	0.080	0.120	0.039	0.192	0.569
48	Revista de Enfermagem e Atenção a Saùde	12	3.0%<br>12	0.043	0.394	0.425	0.253	0.146	0.304	0.832	0.331	0.955
49	International Journal of Nursing Studies	64	16.2%<br>64	0.031	0.305	0.565	0.089	0.428	0.995	0.060	0.573	0.139
50	Journal of Nursing Scholarship	38	9.6%<br>38	0.044	0.382	0.852	0.181	0.544	0.057	0.159	0.267	0.000
51	European Journal of Cardiovascular Nursing	26	6.6%<br>26	0.852	0.994	0.729	0.017	0.024	0.047	0.031	0.188	0.752
52	Nursing Outlook	17	4.3%<br>17	0.034	0.339	0.606	0.205	0.594	0.213	0.245	0.070	0.001
53	European Journal of Cancer Care	19	4.8%<br>19	0.591	0.953	0.894	0.198	0.063	0.001	0.114	0.150	0.091
54	Birth-issues in perinatal care	8	2.0%<br>8	0.165	0.582	0.165	0.259	0.350	0.517	0.256	0.481	0.887
55	Journal of advanced Nursing	57	14.5%<br>57	0.081	0.490	0.641	0.084	0.067	0.003	0.008	0.461	0.002
56	Worldviews on Evidence-Based Nursing	17	4.3%<br>17	0.353	0.848	0.799	0.789	0.611	0.270	0.467	0.042	0.025
57	Journal of Cardiovascular Nursing	29	7.4%<br>29	0.913	0.990	0.455	0.205	0.561	0.279	0.323	0.095	0.687
58	Nurse Education Today	32	8.1%<br>32	0.056	0.437	0.793	0.199	0.228	0.042	0.164	0.045	0.000

59	American Journal of Critical Care	27	6.9%<br>27	0.392	0.915	0.319	0.703	0.223	0.026	0.595	0.220	0.391
60	International Journal of Mental Health Nursing	26	6.6%<br>26	0.531	0.947	0.843	0.579	0.143	0.374	0.230	0.029	0.024
61	Journal of Family Nursing	24	6.1%24	0.741	0.992	0.873	0.558	0.203	0.323	0.449	0.104	0.000
62	Australian Critical Care	13	3.3%<br>13	0.656	0.983	0.639	0.939	0.749	0.786	0.410	0.023	0.001
63	Journal of Tissue Viability	8	2.0%<br>8	0.696	0.985	0.079	0.486	0.726	0.847	0.031	0.824	0.042
64	Journal of Nursing Management	28	7.1%<br>28	0.783	0.995	0.813	0.217	0.326	0.232	0.401	0.112	0.006
65	Nursing Ethics	13	3.3%<br>13	0.269	0.830	0.206	0.307	0.294	0.302	0.338	0.038	0.001
66	Cancer Nursing	10	2.5%<br>10	0.539	0.960	0.433	0.115	0.028	0.016	0.408	0.002	0.065
67	Journal of Human Lactation	7	1.8%<br>7	0.147	0.531	0.248	0.689	0.329	0.630	0.783	0.318	0.721
68	Women and Birth	11	2.8%<br>11	0.066	0.501	0.301	0.898	0.392	0.943	0.954	0.005	0.273
69	World Psychiatry	8	2.0%<br>8	0.575	0.979	0.794	0.542	0.230	0.576	0.224	0.960	0.267
70	Diabetes Care	27	6.9%<br>27	0.392	0.915	0.179	0.202	0.872	0.870	0.556	0.301	0.178
71	Stroke	8	2.0%<br>8	0.696	0.985	0.896	0.144	0.270	0.951	0.731	0.000	0.000
72	American Journal of Clinical Nutrition	22	5.6%<br>22	0.878	0.981	0.405	0.763	0.656	0.688	0.667	0.865	0.257
73	International Journal of Obesity	17	4.3%<br>17	0.202	0.717	0.799	0.154	0.469	0.930	0.441	0.772	0.532
74	Resucitation	10	2.5%<br>10	0.214	0.770	0.387	0.933	0.570	0.883	0.626	0.003	0.000

75	International Journal of Behavioral Nutrition and Physical Activity	10	2.5%<br>10	0.632	0.979	0.745	0.127	0.210	0.619	0.665	0.008	0.665
76	Nutrition Reviews	11	2.8%<br>11	0.923	0.999	0.301	0.404	0.536	0.585	0.525	0.819	0.525
77	Current Opinion in HIV and AIDS	6	1.5%<br>6	0.850	0.999	0.930	0.929	0.830	0.960	0.081	0.000	0.081
78	Advances in Nutrition	11	2.8%<br>11	0.733	0.995	0.516	0.869	0.259	0.491	0.224	0.012	0.224
79	Journal of the American Medical Directors Association	5	1.3%<br>5	0.553	0.952	0.912	0.707	0.836	0.787	0.423	0.000	0.423
80	Journal of Pain and Symptom Management	13	3.3%<br>13	0.638	0.981	0.249	0.509	0.995	0.786	0.856	0.015	0.856
81	Journal of Palliative Medicine	19	4.8%<br>19	0.339	0.884	0.750	0.403	0.138	0.799	0.514	0.071	0.514
82	Patient	4	1.0%<br>4	0.464	0.956	0.979	0.131	0.629	0.085	0.595	0.863	0.676

4. Contrast of hypotheses, demographic variables, and responses to ‘knowing journals in Spanish, Portuguese, and English’.

Order	Journals	Total	Percentage Total	Language*	Country*	Sex*	Experience* (years)	Age* (years)	Situation* working/studying	Public work* Private work	Primary care* Hospital care	Job profile*
1	Index de Enfermería*	109	27.7% 109	0.000	0.000	0.043	0.245	0.333	0.720	0.104	0.111	0.218
2	Investigación y Educación en Enfermería	91	23.1% 91	0.007	0.048	0.216	0.977	0.862	0.391	0.815	0.004	0.008
3	Aquichán	36	9.1% 36	0.085	0.129	0.020	0.087	0.002	0.003	0.012	0.200	0.000
4	Enfermería Intensiva	91	32.1% 91	0.002	0.049	0.045	0.064	0.006	0.009	0.103	0.319	0.001
5	Enfermería universitaria	56	14.2% 56	0.039	0.345	0.759	0.620	0.545	0.601	0.754	0.653	0.443
6	Enfermería Global	91	23.1% 91	0.011	0.076	0.005	0.013	0.021	0.012	0.046	0.166	0.083
7	Enfermería Nefrológica	78	19.8% 78	0.000	0.000	0.091	0.008	0.008	0.000	0.090	0.010	0.001
8	Revista ENE de Enfermería	86	21.8% 86	0.000	0.001	0.061	0.195	0.027	0.049	0.640	0.010	0.000
9	Revista CUIDARTE	114	28.9% 114	0.029	0.222	0.346	0.641	0.497	0.043	0.221	0.047	0.135
10	Avances en Enfermería	88	22.3% 88	0.001	0.027	0.003	0.085	0.020	0.003	0.002	0.008	0.010
11	Enfermería Clínica	99	25.1% 99	0.000	0.010	0.021	0.012	0.000	0.001	0.017	0.047	0.032
12	Cultura de los Cuidados	75	19% 75	0.004	0.029	0.007	0.129	0.071	0.025	0.128	0.001	0.200

13	Investigación en enfermería: imagen y desarrollo	52	13.2%<br>52	0.153	0.162	0.478	0.142	0.070	0.874	0.969	0.028	0.776
14	Temperamentvm	34	8.6%<br>34	0.007	0.130	0.037	0.048	0.006	0.003	0.017	0.632	0.040
15	Gerokomos	60	15.2%<br>60	0.000	0.000	0.007	0.001	0.000	0.000	0.000	0.013	0.000
16	Revista Ética de los Cuidados	70	17.8%<br>70	0.045	0.112	0.017	0.039	0.001	0.000	0.002	0.096	0.002
17	Archivos de la Memoria	30	7.6%<br>30	0.016	0.195	0.004	0.216	0.073	0.035	0.048	0.729	0.372
18	Revista Tesela	42	10.7%<br>42	0.001	0.025	0.000	0.000	0.000	0.000	0.029	0.085	0.000
19	Revista Cubana de Enfermería	71	18%<br>71	0.027	0.133	0.638	0.697	0.342	0.056	0.091	0.080	0.009
20	Revista de Enfermería del Instituto Mexicano del Seguro Social	24	6.1%<br>24	0.140	0.082	0.907	0.199	0.269	0.494	0.504	0.053	0.002
21	Metas de Enfermería	81	2.6%<br>81	0.000	0.002	0.009	0.001	0.000	0.000	0.002	0.170	0.003
22	Enfermería Comunitaria. Revista internacional de cuidados de salud familiar y comunitaria	93	23.6%<br>93	0.001	0.027	0.181	0.012	0.012	0.001	0.007	0.264	0.217
23	Matronas Profesión	46	11.7%<br>46	0.000	0.001	0.017	0.004	0.002	0.003	0.118	0.280	0.005
24	Revista Rol de Enfermería	102	25.9%<br>102	0.000	0.000	0.047	0.000	0.000	0.000	0.012	0.001	0.001
25	Enfermería Docente	64	16.5%<br>64	0.000	0.000	0.059	0.004	0.002	0.001	0.114	0.174	0.000
26	Biblioteca Lascasas	41	10.4%<br>41	0.001	0.004	0.001	0.000	0.000	0.000	0.002	0.482	0.000
27	Revista da Escola de Enfermagem da USP	79	20.1%<br>79	0.000	0.000	0.315	0.072	0.763	0.044	0.726	0.007	0.001

28	Texto & Contexto: Enfermagem	82	20.8%<br>82	0.007	0.017	0.327	0.539	0.846	0.896	0.053	0.034	0.456
29	Escola Anna Nery Revista de Enfermagem	50	12.7%<br>50	0.000	0.000	0.724	0.398	0.085	0.006	0.069	0.113	0.105
30	Revista Latino-Americana de Enfermagem	80	20.3%<br>80	0.000	0.000	0.119	0.384	0.917	0.182	0.283	0.092	0.011
31	Brasileira de Enfermagem	79	20.1%<br>79	0.000	0.000	0.603	0.038	0.157	0.001	0.330	0.084	0.096
32	Revista Gaúcha de Enfermagem	46	11.7%<br>46	0.000	0.000	0.524	0.328	0.176	0.073	0.456	0.001	0.118
33	ACTA Paulista de Enfermagem	52	13.5%<br>52	0.000	0.000	0.310	0.392	0.158	0.005	0.548	0.002	0.023
34	Ciência, Cuidado e Saúde	69	17.5%<br>69	0.000	0.000	0.425	0.166	0.218	0.001	0.652	0.065	0.509
35	Revista Eletrônica de Enfermagem	72	18.3%<br>72	0.000	0.000	0.694	0.375	0.480	0.110	0.733	0.018	0.078
36	Revista da Rede de Enfermagem do Nordeste	30	7.6%<br>30	0.000	0.000	0.178	0.671	0.069	0.003	0.934	0.004	0.405
37	Cogitare Enfermagem	39	9.9%<br>39	0.000	0.000	0.392	0.234	0.174	0.016	0.636	0.012	0.486
38	Revista de Enfermagem da UFSM	37	9.34%<br>37	0.000	0.000	0.181	0.032	0.018	0.028	0.666	0.000	0.382
39	Revista Mineira de Enfermagem	34	8.6%<br>34	0.000	0.000	0.309	0.232	0.081	0.012	0.888	0.000	0.235
40	Revista Enfermagem em Foco	46	11.7%<br>46	0.000	0.000	0.363	0.050	0.002	0.000	0.368	0.029	0.096
41	Revista SOBECC	33	8.4%<br>33	0.000	0.000	0.309	0.217	0.174	0.015	0.449	0.040	0.007
42	Revista de Enfermagem Referência	36	9.1%<br>36	0.000	0.000	0.354	0.406	0.718	0.101	0.817	0.102	0.121
43	Revista de Enfermagem do Centro-Oeste Mineiro	20	5.1%<br>20	0.000	0.000	0.093	0.092	0.688	0.111	0.547	0.019	0.258

44	Revista de Enfermagem UFPE On Line	26	6.6%<br>26	0.000	0.000	0.814	0.087	0.075	0.004	0.943	0.008	0.743
45	Revista Baiana de Enfermagem	26	6.6%<br>26	0.000	0.000	0.813	0.082	0.006	0.100	0.948	0.091	0.922
46	Revista de Enfermagem da Universidade Federal do Piauí	26	6.6%<br>26	0.000	0.000	0.893	0.064	0.051	0.001	0.713	0.008	0.560
47	Revista de Pesquisa: Cuidado é Fundamental	36	9.1%<br>36	0.000	0.000	0.354	0.503	0.083	0.046	0.377	0.001	0.145
48	Revista de Enfermagem e Atenção a Saùde	37	9.4%<br>37	0.000	0.000	0.835	0.960	0.665	0.055	0.782	0.013	0.328
49	International Journal of Nursing Studies	126	32%<br>126	0.015	0.100	0.319	0.957	0.438	0.618	0.039	0.357	0.196
50	Journal of Nursing Scholarship	68	17.3%<br>68	0.011	0.044	0.296	0.344	0.333	0.044	0.095	0.046	0.003
51	European Journal of Cardiovascular Nursing	78	19.8%<br>78	0.101	0.111	0.019	0.875	0.665	0.043	0.612	0.134	0.110
52	Nursing Outlook	50	12.7%<br>50	0.068	0.452	0.340	0.238	0.093	0.041	0.003	0.037	0.000
53	European Journal of Cancer Care	59	15%<br>59	0.042	0.089	0.182	0.277	0.017	0.101	0.201	0.060	0.000
54	Birth-issues in perinatal care	21	5.3%<br>21	0.145	0.004	0.252	0.471	0.086	0.299	0.195	0.066	0.211
55	Journal of advanced Nursing	97	24.6%<br>97	0.780	0.235	0.095	0.142	0.226	0.005	0.001	0.201	0.010
56	Worldviews on Evidence-Based Nursing	40	10.2%<br>40	0.214	0.097	0.108	0.066	0.027	0.045	0.009	0.003	0.003
57	Journal of Cardiovascular Nursing	61	15.5%<br>61	0.004	0.013	0.036	0.496	0.826	0.453	0.503	0.021	0.449
58	Nurse Education Today	52	13.2%<br>52	0.065	0.087	0.051	0.241	0.130	0.056	0.063	0.010	0.000
59	American Journal of Critical Care	69	17.5%<br>69	0.000	0.016	0.652	0.034	0.008	0.002	0.023	0.338	0.000

60	International Journal of Mental Health Nursing	62	15.7% 62	0.035	0.308	0.414	0.711	0.687	0.127	0.052	0.247	0.135
61	Journal of Family Nursing	52	13.2%52	0.038	0.344	0.070	0.008	0.002	0.006	0.001	0.502	0.002
62	Australian Critical Care	30	7.6% 30	0.028	0.311	0.001	0.291	0.345	0.012	0.002	0.001	0.000
63	Journal of Tissue Viability	15	3.8% 15	0.194	0.777	0.015	0.568	0.369	0.526	0.022	0.037	0.000
64	Journal of Nursing Management	40	10.2% 40	0.034	0.338	0.072	0.061	0.148	0.098	0.078	0.461	0.000
65	Nursing Ethics	46	11.7% 46	0.015	0.203	0.012	0.186	0.049	0.026	0.003	0.119	0.000
66	Cancer Nursing	33	8.4% 33	0.046	0.402	0.304	0.724	0.783	0.898	0.582	0.002	0.362
67	Journal of Human Lactation	40	10.2% 40	0.034	0.336	0.412	0.332	0.114	0.929	0.718	0.061	0.441
68	Women and Birth	18	4.6% 18	0.140	0.613	0.799	0.248	0.172	0.107	0.006	0.000	0.003
69	World Psychiatry	24	6.1% 24	0.145	0.006	0.738	0.706	0.930	0.733	0.135	0.000	0.286
70	Diabetes Care	79	20.1% 79	0.286	0.403	0.683	0.540	0.783	0.213	0.502	0.189	0.076
71	Stroke	33	8.4% 33	0.643	0.104	0.708	0.726	0.489	0.316	0.116	0.000	0.041
72	American Journal of Clinical Nutrition	56	14.2% 56	0.161	0.672	0.154	0.538	0.047	0.079	0.055	0.025	0.102
73	International Journal of Obesity	41	10.4% 41	0.196	0.073	0.029	0.279	0.209	0.270	0.519	0.122	0.927
74	Resucitation	31	7.9% 31	0.684	0.098	0.011	0.186	0.045	0.124	0.157	0.000	0.585
75	International Journal of Behavioral Nutrition and Physical Activity	31	7.9% 31	0.197	0.036	0.121	0.233	0.101	0.615	0.037	0.001	0.878

76	Nutrition Reviews	35	8.9%<br>35	0.177	0.056	0.035	0.301	0.624	0.219	0.128	0.498	0.005
77	Current Opinion in HIV and AIDS	18	4.6%<br>18	0.145	0.637	0.087	0.041	0.119	0.354	0.411	0.081	0.732
78	Advances in Nutrition	34	8.6%<br>34	0.411	0.921	0.295	0.757	0.501	0.540	0.563	0.005	0.000
79	Journal of the American Medical Directors Association	22	5.6%<br>22	0.484	0.013	0.586	0.647	0.699	0.240	0.078	0.161	0.947
80	Journal of Pain and Symptom Management	30	7.6%<br>30	0.155	0.021	0.095	0.075	0.156	0.189	0.003	0.001	0.004
81	Journal of Palliative Medicine	38	9.6%<br>38	0.513	0.159	0.273	0.055	0.361	0.148	0.003	0.001	0.033
82	Patient	23	5.8%<br>23	0.283	0.012	0.486	0.393	0.165	0.152	0.047	0.814	0.007

5. Contrast of hypotheses, demographic variables, and responses to ‘not knowing journals in Spanish, Portuguese, and English’.

Order	Not knowing journals	Total	Percentage Total	Language*	Country*	Sex*	Experience* (years)	Age* (years)	Situation* working/studying	Public work* Private work	Primary care* Hospital care	Job profile*
1	Index de Enfermería*	302	76.6% 302	0.000	0.000	0.442	0.002	0.000	0.000	0.193	0.454	0.026
2	Investigación y Educación en Enfermería	124	31.5% 124	0.039	0.187	0.462	0.786	0.467	0.010	0.836	0.434	0.198
3	Aquichán	251	63.7% 251	0.000	0.001	0.518	0.035	0.047	0.000	0.022	0.545	0.000
4	Enfermería Intensiva	160	40.6% 160	0.000	0.002	0.140	0.006	0.000	0.000	0.582	0.773	0.001
5	Enfermería universitaria	211	53.6% 211	0.009	0.050	0.456	0.696	0.975	0.699	0.360	0.583	0.294
6	Enfermería Global	142	36% 142	0.000	0.000	0.379	0.049	0.006	0.000	0.064	0.392	0.002
7	Enfermería Nefrológica	185	47% 185	0.000	0.000	0.196	0.002	0.002	0.000	0.462	0.616	0.167
8	Revista ENE de Enfermería	166	42.1% 166	0.000	0.000	0.203	0.020	0.002	0.000	0.288	0.230	0.001
9	Revista CUIDARTE	131	33.2% 131	0.001	0.005	0.991	0.042	0.024	0.000	0.547	0.047	0.084
10	Avances en Enfermería	168	42.6% 168	0.000	0.000	0.510	0.002	0.000	0.000	0.005	0.302	0.018
11	Enfermería Clínica	124	31.5% 124	0.000	0.000	0.606	0.000	0.000	0.000	0.040	0.154	0.005

12	Cultura de los Cuidados	190	48.2%<br>190	0.000	0.000	0.425	0.015	0.005	0.000	0.216	0.211	0.172
13	Investigación en enfermería: imagen y desarrollo	232	58.9%<br>232	0.003	0.012	0.963	0.054	0.126	0.210	0.744	0.635	0.122
14	Temperamentvm	265	67.3%<br>265	0.000	0.000	0.227	0.018	0.006	0.000	0.138	0.242	0.223
15	Gerokomos	221	56.1%<br>221	0.000	0.000	0.068	0.000	0.000	0.000	0.002	0.013	0.000
16	Revista Ética de los Cuidados	202	51.3%<br>202	0.000	0.001	0.331	0.000	0.000	0.000	0.063	0.433	0.003
17	Archivos de la Memoria	256	65%<br>256	0.000	0.000	0.716	0.039	0.225	0.006	0.617	0.835	0.354
18	Revista Tesela	247	62.7%<br>247	0.000	0.000	0.083	0.000	0.000	0.000	0.405	0.315	0.103
19	Revista Cubana de Enfermería	191	48.5%<br>191	0.000	0.000	0.834	0.540	0.338	0.003	0.256	0.871	0.316
20	Revista de Enfermería del Instituto Mexicano del Seguro Social	269	68.3%<br>269	0.000	0.000	0.540	0.458	0.787	0.775	0.611	0.407	0.039
21	Metas de Enfermería	170	43.1%<br>170	0.000	0.000	0.283	0.000	0.000	0.000	0.003	0.022	0.000
22	Enfermería Comunitaria. Revista internacional de cuidados de salud familiar y comunitaria	150	38.1%<br>150	0.000	0.000	0.183	0.000	0.000	0.000	0.037	0.001	0.005
23	Matronas Profesión	247	62.7%<br>247	0.000	0.000	0.984	0.009	0.049	0.002	0.279	0.389	0.186
24	Revista Rol de Enfermería	141	35.8%<br>141	0.000	0.000	0.063	0.000	0.000	0.000	0.005	0.000	0.000
25	Enfermería Docente	213	54.1%<br>213	0.000	0.000	0.607	0.001	0.003	0.000	0.365	0.899	0.004
26	Biblioteca Lascasas	244	61.9%<br>244	0.000	0.000	0.194	0.004	0.001	0.000	0.143	0.792	0.001

27	Revista da Escola de Enfermagem da USP	184	46.7%<br>184	0.000	0.000	0.121	0.563	0.363	0.228	0.667	0.000	0.000
28	Texto & Contexto: Enfermagem	178	45.2%<br>178	0.000	0.000	0.251	0.140	0.187	0.824	0.339	0.008	0.065
29	Escola Anna Nery Revista de Enfermagem	228	57.9%<br>228	0.000	0.000	0.025	0.085	0.120	0.015	0.379	0.002	0.001
30	Revista Latino-Americana de Enfermagem	174	44.2%<br>174	0.000	0.000	0.381	0.371	0.607	0.769	0.668	0.004	0.002
31	Brasileira de Enfermagem	179	45.4%<br>179	0.000	0.000	0.024	0.585	0.371	0.005	0.686	0.004	0.001
32	Revista Gaúcha de Enfermagem	230	58.4%<br>230	0.000	0.000	0.029	0.038	0.016	0.147	0.510	0.007	0.006
33	ACTA Paulista de Enfermagem	215	54.6%<br>215	0.000	0.000	0.109	0.308	0.064	0.021	0.715	0.000	0.000
34	Ciência, Cuidado e Saúde	202	51.3%<br>202	0.000	0.000	0.042	0.119	0.068	0.007	0.447	0.001	0.008
35	Revista Eletrônica de Enfermagem	198	50.3%<br>198	0.000	0.000	0.012	0.417	0.167	0.005	0.576	0.000	0.000
36	Revista da Rede de Enfermagem do Nordeste	250	63.5%<br>250	0.000	0.000	0.029	0.103	0.053	0.021	0.924	0.001	0.001
37	Cogitare Enfermagem	242	61.4%<br>242	0.000	0.000	0.019	0.243	0.298	0.076	0.666	0.003	0.003
38	Revista de Enfermagem da UFSM	244	61.9%<br>244	0.000	0.000	0.112	0.092	0.051	0.133	0.795	0.000	0.002
39	Revista Mineira de Enfermagem	249	63.2%<br>249	0.000	0.000	0.014	0.041	0.094	0.008	0.778	0.000	0.000
40	Revista Enfermagem em Foco	237	60.2%<br>237	0.000	0.000	0.001	0.037	0.055	0.000	0.855	0.001	0.000
41	Revista SOBECC	257	65.2%<br>257	0.000	0.000	0.025	0.029	0.042	0.016	0.319	0.005	0.005
42	Revista de Enfermagem Referência	252	64%<br>252	0.000	0.000	0.016	0.529	0.704	0.186	0.557	0.003	0.008

43	Revista de Enfermagem do Centro-Oeste Mineiro	275	69.8%<br>275	0.000	0.000	0.011	0.155	0.091	0.177	0.684	0.006	0.015
44	Revista de Enfermagem UFPE On Line	260	66%<br>260	0.000	0.000	0.039	0.011	0.015	0.038	0.905	0.002	0.004
45	Revista Baiana de Enfermagem	257	65.2%<br>257	0.000	0.000	0.016	0.045	0.021	0.104	0.850	0.009	0.007
46	Revista de Enfermagem da Universidade Federal do Piauí	268	68%<br>268	0.000	0.000	0.120	0.012	0.015	0.007	0.730	0.005	0.009
47	Revista de Pesquisa: Cuidado é Fundamental	249	63.2%<br>249	0.000	0.000	0.029	0.100	0.032	0.216	0.369	0.007	0.016
48	Revista de Enfermagem e Atenção a Saùde	248	62.9%<br>248	0.000	0.000	0.055	0.406	0.324	0.130	0.962	0.001	0.046
49	International Journal of Nursing Studies	90	22.8%<br>90	0.076	0.070	0.227	0.018	0.138	0.560	0.388	0.893	0.381
50	Journal of Nursing Scholarship	174	44.2%<br>174	0.049	0.116	0.528	0.210	0.306	0.028	0.844	0.110	0.008
51	European Journal of Cardiovascular Nursing	161	40.9%<br>161	0.033	0.109	0.179	0.488	0.074	0.293	0.476	0.184	0.039
52	Nursing Outlook	201	51%<br>201	0.005	0.019	0.644	0.016	0.069	0.047	0.875	0.505	0.034
53	European Journal of Cancer Care	187	47.5%<br>187	0.002	0.012	0.799	0.244	0.039	0.825	0.853	0.725	0.185
54	Birth-issues in perinatal care	238	60.4%<br>238	0.001	0.001	0.652	0.154	0.455	0.902	0.608	0.467	0.282
55	Journal of advanced Nursing	135	34.3%<br>135	0.003	0.030	0.954	0.384	0.268	0.007	0.228	0.192	0.003
56	Worldviews on Evidence-Based Nursing	216	54.8%<br>216	0.000	0.002	0.607	0.024	0.054	0.030	0.568	0.127	0.027
57	Journal of Cardiovascular Nursing	188	47.7%<br>188	0.003	0.013	0.712	0.135	0.219	0.904	0.602	0.123	0.040
58	Nurse Education Today	190	48.2%<br>190	0.006	0.024	0.614	0.006	0.021	0.011	0.743	0.669	0.003

59	American Journal of Critical Care	161	40.9% 161	0.069	0.186	0.861	0.117	0.057	0.095	0.141	0.175	0.024
60	International Journal of Mental Health Nursing	163	41.4% 163	0.612	0.623	0.333	0.676	0.337	0.674	0.739	0.044	0.165
61	Journal of Family Nursing	194	49.2%194	0.132	0.182	0.792	0.027	0.005	0.148	0.361	0.156	0.046
62	Australian Critical Care	232	58.9% 232	0.049	0.047	0.865	0.124	0.238	0.210	0.394	0.111	0.255
63	Journal of Tissue Viability	248	62.9% 248	0.013	0.012	0.552	0.068	0.295	0.974	0.477	0.573	0.536
64	Journal of Nursing Management	212	53.8% 212	0.013	0.031	0.633	0.036	0.066	0.227	0.639	0.178	0.012
65	Nursing Ethics	208	52.8% 208	0.108	0.134	0.887	0.017	0.037	0.104	0.747	0.817	0.054
66	Cancer Nursing	216	54.8% 216	0.025	0.049	0.375	0.220	0.616	0.979	0.643	0.185	0.574
67	Journal of Human Lactation	250	63.5% 250	0.000	0.002	0.598	0.196	0.244	0.809	0.916	0.286	0.167
68	Women and Birth	241	61.2% 241	0.028	0.045	0.372	0.075	0.193	0.240	0.381	0.589	0.336
69	World Psychiatry	232	58.9% 232	0.036	0.025	0.923	0.500	0.836	0.785	0.397	0.179	0.058
70	Diabetes Care	157	39.8% 157	0.155	0.255	0.813	0.756	0.435	0.826	0.578	0.518	0.308
71	Stroke	224	56.9% 224	0.064	0.044	0.869	0.289	0.451	0.481	0.808	0.638	0.705
72	American Journal of Clinical Nutrition	188	47.7% 188	0.018	0.039	0.343	0.240	0.113	0.144	0.317	0.367	0.175
73	International Journal of Obesity	207	52.5% 207	0.002	0.007	0.711	0.088	0.070	0.820	0.278	0.531	0.363
74	Resucitation	222	56.3% 222	0.021	0.034	0.884	0.498	0.327	0.291	0.673	0.728	0.352

75	International Journal of Behavioral Nutrition and Physical Activity	235	59.6%<br>235	0.002	0.003	0.840	0.012	0.042	0.489	0.659	0.160	0.179
76	Nutrition Reviews	230	58.4%<br>230	0.005	0.008	0.611	0.169	0.517	0.486	0.488	0.246	0.032
77	Current Opinion in HIV and AIDS	247	62.7%<br>247	0.008	0.010	0.756	0.700	0.402	0.693	0.576	0.246	0.180
78	Advances in Nutrition	228	57.9%<br>228	0.023	0.035	0.940	0.552	0.713	0.573	0.884	0.264	0.013
79	Journal of the American Medical Directors Association	243	61.7%<br>243	0.005	0.006	0.741	0.111	0.268	0.592	0.922	0.176	0.323
80	Journal of Pain and Symptom Management	234	59.4%<br>234	0.004	0.006	0.382	0.115	0.123	0.238	0.434	0.070	0.101
81	Journal of Palliative Medicine	209	53%<br>209	0.132	0.095	0.580	0.035	0.587	0.283	0.403	0.309	0.502
82	Patient	243	61.7%<br>243	0.016	0.009	0.571	0.543	0.429	0.692	0.595	0.039	0.224
