# Supplementary material for: Research, Reading, and Publication Habits of Nurses and Nursing Students Applied to Impact Journals: International Multicentre Study
Source: Int J Environ Res Public Health. 2023 Mar 7;20(6):4697. doi: 10.3390/ijerph20064697 (PMC10049027; doi:10.3390/ijerph20064697)
Supplement: Supplementary file 1 [file ijerph-20-04697-s001.zip › Table S4. 1Q_LcEE-CAPC Questionnaire. Reading, referencing, publishing.pdf]

Table S4. 1Q_LcEE-CAPC Questionnaire. Responses to 'reading, referencing, publishing in, knowing, and not knowing journals in Spanish, Portuguese, and English'

Order	Journals	Reading	Referencing	Publishing	Knowing	Not knowing
1	Index de Enfermería*	45.7%<br>180	21.1%<br>83	5.1%<br>20	27.7%<br>109	76.6%<br>302
2	Investigación y Educación en Enfermería	32%<br>126	10.4%<br>41	1.3%<br>5	23.1%<br>91	31.5%<br>124
3	Aquichán	8.9%<br>35	6.1%<br>24	1.5%<br>6	9.1%<br>36	63.7%<br>251
4	Enfermería Intensiva	23.4%<br>92	7.6%<br>30	2.8%<br>11	32.1%<br>91	40.6%<br>160
5	Enfermería universitaria	14.5%<br>57	3.8%<br>15	1%<br>4	14.2%<br>56	53.6%<br>211
6	Enfermería Global	29.7%<br>117	17.8%<br>70	4.6%<br>18	23.1%<br>91	36%<br>142
7	Enfermería Nefrológica	16.8%<br>66	5.8%<br>23	1.3%<br>5	19.8%<br>78	47%<br>185
8	Revista ENE de Enfermería	25.4%<br>100	10.9%<br>43	4.6%<br>18	21.8%<br>86	42.1%<br>166
9	Revista CUIDARTE	25.1%<br>99	7.6%<br>30	0.3%<br>1	28.9%<br>114	33.2%<br>131
10	Avances en Enfermería	18.8%<br>74	6.6%<br>26	0.5%<br>2	22.3%<br>88	42.6%<br>168
11	Enfermería Clínica	37.6%<br>148	19.5%<br>77	7.9%<br>31	25.1%<br>99	31.5%<br>124
12	Cultura de los Cuidados	22.1%<br>87	8.6%<br>34	3.3%<br>13	19%<br>75	48.2%<br>190
13	Investigación en enfermería: imagen y desarrollo	9.6%<br>38	3.6%<br>14	0.5%<br>2	13.2%<br>52	58.9%<br>232
14	Temperamentvm	7.4%<br>29	3.3%<br>13	1.3%<br>5	8.6%<br>34	67.3%<br>265
15	Gerokomos	16.2%<br>64	8.4%<br>33	2.5%<br>10	15.2%<br>60	56.1%<br>221
16	Revista Ética de los Cuidados	15.2%<br>60	3.3%<br>13	1.5%<br>6	17.8%<br>70	51.3%<br>202
17	Archivos de la Memoria	7.6%<br>30	2.0%<br>8	1.3%<br>5	7.6%<br>30	65%<br>256
18	Revista Tesela	7.6%<br>30	2.0%<br>8	0.8<br>3	10.7%<br>42	62.7%<br>247
19	Revista Cubana de Enfermería	21.3%<br>84	21.3%<br>84	2%<br>8	18%<br>71	48.5%<br>191
20	Revista de Enfermería del Instituto Mexicano del Seguro Social	5.8%<br>23	5.8%<br>23	0.3%<br>1	6.1%<br>24	68.3%<br>269
21	Metas de Enfermería	31.0%<br>122	31.0%<br>122	5.3%<br>21	2.6%<br>81	43.1%<br>170
22	Enfermería Comunitaria. Revista internacional de cuidados de salud familiar y comunitaria	29.7%<br>117	29.7%<br>117	2.3%<br>9	23.6%<br>93	38.1%<br>150
23	Matronas Profesión	7.1%<br>28	7.1%<br>28	1%<br>4	11.7%<br>46	62.7%<br>247
24	Revista Rol de Enfermería	34.3%<br>135	34.3%<br>135	6.1%<br>24	25.9%<br>102	35.8%<br>141
25	Enfermería Docente	12.2%<br>48	12.2%<br>48	1.5%<br>6	16.5%<br>64	54.1%<br>213
26	Biblioteca Lascasas	10.4%<br>41	10.4%<br>41	1.3%<br>5	10.4%<br>41	61.9%<br>244
27	Revista da Escola de Enfermagem da USP	20.6%<br>81	13.2%<br>52	4.6%<br>18	20.1%<br>79	46.7%<br>184
28	Texto & Contexto: Enfermagem	19.8%<br>78	13.2%<br>52	2.3%<br>9	20.8%<br>82	45.2%<br>178
29	Escola Anna Nery Revista de Enfermagem	12.7%<br>50	8.1%<br>32	1.8%<br>7	12.7%<br>50	57.9%<br>228
30	Revista Latino-Americana de Enfermagem	23.6%<br>93	14.0%<br>55	3.8%<br>15	20.3%<br>80	44.2%<br>174
31	Brasileira de Enfermagem	20.8%<br>82	14.0%<br>55	4.6%<br>18	20.1%<br>79	45.4%<br>179
32	Revista Gaúcha de Enfermagem	14.0%<br>55	10.4%<br>41	2%<br>8	11.7%<br>46	58.4%<br>230
33	ACTA Paulista de Enfermagem	16.0%<br>63	10.2%<br>40	1.8%<br>7	13.5%<br>52	54.6%<br>215
34	Ciência, Cuidado e Saúde	16.2%<br>64	7.9%<br>31	2.5%<br>10	17.5%<br>69	51.3%<br>202

35	Revista Eletrônica de Enfermagem	18.0%<br>71	10.2%<br>40	2.5%<br>10	18.3%<br>72	50.3%<br>198
36	Revista da Rede de Enfermagem do Nordeste	10.7%<br>42	6.3%<br>25	3.8%<br>15	7.6%<br>30	63.5%<br>250
37	Cogitare Enfermagem	10.4%<br>41	6.6%<br>26	1.5%<br>6	9.9%<br>39	61.4%<br>242
38	Revista de Enfermagem da UFSM	10.9%<br>43	6.1%<br>24	1.3%<br>5	9.34%<br>37	61.9%<br>244
39	Revista Mineira de Enfermagem	10.9%<br>43	5.1%<br>20	1.5%<br>6	8.6%<br>34	63.2%<br>249
40	Revista Enfermagem em Foco	11.4%<br>45	4.6%<br>18	2%<br>8	11.7%<br>46	60.2%<br>237
41	Revista SOBECC	6.6%<br>26	1.3%<br>5	0.8%<br>3	8.4%<br>33	65.2%<br>257
42	Revista de Enfermagem Referência	7.9%<br>31	4.8%<br>19	1.5%<br>6	9.1%<br>36	64%<br>252
43	Revista de Enfermagem do Centro-Oeste Mineiro	3.6%<br>14	2.0%<br>8	1%<br>4	5.1%<br>20	69.8%<br>275
44	Revista de Enfermagem UFPE On Line	9.4%<br>37	7.4%<br>29	6.1%<br>24	6.6%<br>26	66%<br>260
45	Revista Baiana de Enfermagem	9.1%<br>36	4.6%<br>18	2.8%<br>11	6.6%<br>26	65.2%<br>257
46	Revista de Enfermagem da Universidade Federal do Piauí	5.8%<br>23	2.5%<br>10	0.5%<br>2	6.6%<br>26	68%<br>268
47	Revista de Pesquisa: Cuidado é Fundamental	8.9%<br>35	6.1%<br>24	2.5%<br>10	9.1%<br>36	63.2%<br>249
48	Revista de Enfermagem e Atenção a Saùde	7.6%<br>30	3.0%<br>12	0.3%<br>1	9.4%<br>37	62.9%<br>248
49	International Journal of Nursing Studies	32.5%<br>128	16.2%<br>64	0.8%<br>3	32%<br>126	22.8%<br>90
50	Journal of Nursing Scholarship	15.2%<br>60	9.6%<br>38	1.3%<br>5	17.3%<br>68	44.2%<br>174
51	European Journal of Cardiovascular Nursing	13.5%<br>53	6.6%<br>26	0.5%<br>2	19.8%<br>78	40.9%<br>161
52	Nursing Outlook	11.7%<br>46	4.3%<br>17	0.8%<br>3	12.7%<br>50	51%<br>201
53	European Journal of Cancer Care	9.9%<br>39	4.8%<br>19	0.3%<br>1	15%<br>59	47.5%<br>187
54	Birth-issues in perinatal care	3.0%<br>12	2.0%<br>8	0.3%<br>1	5.3%<br>21	60.4%<br>238
55	Journal of advanced Nursing	23.4%<br>92	14.5%<br>57	2.8%<br>11	24.6%<br>97	34.3%<br>135
56	Worldviews on Evidence-Based Nursing	6.6%<br>26	4.3%<br>17	0.8%<br>3	10.2%<br>40	54.8%<br>216
57	Journal of Cardiovascular Nursing	10.9%<br>43	7.4%<br>29	0%<br>0	15.5%<br>61	47.7%<br>188
58	Nurse Education Today	14.7%<br>58	8.1%<br>32	2.5%<br>10	13.2%<br>52	48.2%<br>190
59	American Journal of Critical Care	16.2%<br>64	6.9%<br>27	2.5%<br>10	17.5%<br>69	40.9%<br>161
60	International Journal of Mental Health Nursing	15.0%<br>59	6.6%<br>26	4.1%<br>16	15.7%<br>62	41.4%<br>163
61	Journal of Family Nursing	10.2%<br>40	6.1%<br>24	2%<br>8	13.2%<br>52	49.2%<br>194
62	Australian Critical Care	4.3%<br>17	3.3%<br>13	2.3%<br>9	7.6%<br>30	58.9%<br>232
63	Journal of Tissue Viability	2.8%<br>11	2.0%<br>8	0.8%<br>3	3.8%<br>15	62.9%<br>248
64	Journal of Nursing Management	10.9%<br>43	7.1%<br>28	2.8%<br>11	10.2%<br>40	53.8%<br>212
65	Nursing Ethics	8.4%<br>33	3.3%<br>13	1.8%<br>7	11.7%<br>46	52.8%<br>208
66	Cancer Nursing	5.6%<br>22	2.5%<br>10	1.8%<br>7	8.4%<br>33	54.8%<br>216
67	Journal of Human Lactation	2.0%<br>8	1.8%<br>7	0.3%<br>1	10.2%<br>40	63.5%<br>250
68	Women and Birth	4.1%<br>16	2.8%<br>11	0.3%<br>1	4.6%<br>18	61.2%<br>241
69	World Psychiatry	5.1%<br>20	2.0%<br>8	0%<br>0	6.1%<br>24	58.9%<br>232
70	Diabetes Care	17.8%<br>70	6.9%<br>27	0.5%<br>2	20.1%<br>79	39.8%<br>157
71	Stroke	3.8%<br>15	2.0%<br>8	0%<br>0	8.4%<br>33	56.9%<br>224

72	American Journal of Clinical Nutrition	9.1%<br>36	5.6%<br>22	0%<br>0	14.2%<br>56	47.7%<br>188
73	International Journal of Obesity	8.6%<br>34	4.3%<br>17	0.5%<br>2	10.4%<br>41	52.5%<br>207
74	Resucitation	8.4%<br>13	2.5%<br>10	0.3%<br>1	7.9%<br>31	56.3%<br>222
75	International Journal of Behavioral Nutrition and Physical Activity	5.1%<br>20	2.5%<br>10	0.3%<br>1	7.9%<br>31	59.6%<br>235
76	Nutrition Reviews	4.6%<br>18	2.8%<br>11	0%<br>0	8.9%<br>35	58.4%<br>230
77	Current Opinion in HIV and AIDS	2.8%<br>11	1.5%<br>6	0%<br>0	4.6%<br>18	62.7%<br>247
78	Advances in Nutrition	5.3%<br>21	2.8%<br>11	0%<br>0	8.6%<br>34	57.9%<br>228
79	Journal of the American Medical Directors Association	3.3%<br>13	1.3%<br>5	0%<br>0	5.6%<br>22	61.7%<br>243
80	Journal of Pain and Symptom Management	6.1%<br>24	3.3%<br>13	0.3%<br>1	7.6%<br>30	59.4%<br>234
81	Journal of Palliative Medicine	9.6%<br>38	4.8%<br>19	0.3%<br>1	9.6%<br>38	53%<br>209
82	Patient	3.6%<br>14	1.0%<br>4	0%<br>0	5.8%<br>23	61.7%<br>243
