# Supplementary material for: Research, Reading, and Publication Habits of Nurses and Nursing Students Applied to Impact Journals: International Multicentre Study
Source: Int J Environ Res Public Health. 2023 Mar 7;20(6):4697. doi: 10.3390/ijerph20064697 (PMC10049027; doi:10.3390/ijerph20064697)
Supplement: Supplementary file 1 [file ijerph-20-04697-s001.zip › Table S5. Analysis for the creation of impact indexes.pdf]

Table S5. Analysis for the creation of impact indexes and their variability according to the included criteria.

Order	Journals	Ranking Order	Ranking	Ranking Rrp Order	Ranking Rrp	Rankin Knk Order	Rankin Knk	Rk Reading Order	Rk Reading	Reading Total	Reading Percentage	Rk Referencing	Referencing Total	Referencing Percentage	Rk Publishing	Publishing Total	Publishing Percentage	Rk Knowing	Knowing Total	Knowing Percentage	Rk Not Knowing	Rk Not Knowing (Inverse)	Not Knowing Total	Not Knowing Percentage
1	Index de Enfermería*	15	97	82	191	82	191	1	1	180	45.7%<br>180	6	83	21.1%<br>83	5	20	5.1%<br>20	3	109	27.7%<br>109	1	82	302	76.6%<br>302
2	Investigación y Educación en Enfermería	12	76	79	184	35	94	5	5	126	32%<br>126	17	41	10.4%<br>41	41	5	1.3%<br>5	10	91	23.1%<br>91	81	3	124	31.5%<br>124
3	Aquichán	55	259	20	114	73	108	52	52	35	8.9%<br>35	42	24	6.1%<br>24	37	6	1.5%<br>6	56	36	9.1%<br>36	11	72	251	63.7%<br>251
4	Enfermería Intensiva	11	74	41	135	51	101	12	12	92	23.4%<br>92	28	30	7.6%<br>30	15	11	2.8%<br>11	9	91	32.1%<br>91	73	10	160	40.6%<br>160
5	Enfermería universitaria	38	206	71	162	33	93	31	31	57	14.5%<br>57	56	15	3.8%<br>15	49	4	1%<br>4	33	56	14.2%<br>56	46	37	211	53.6%<br>211
6	Enfermería Global	3	36	38	131	40	98	7	7	117	29.7%<br>117	8	70	17.8%<br>70	6	18	4.6%<br>18	8	91	23.1%<br>91	76	7	142	36%<br>142
7	Enfermería Nefrológica	29	152	58	155	46	100	22	22	66	16.8%<br>66	44	23	5.8%<br>23	45	5	1.3%<br>5	19	78	19.8%<br>78	61	22	185	47%<br>185
8	Revista ENE de Enfermería	7	60	31	125	45	100	9	9	100	25.4%<br>100	16	43	10.9%<br>43	9	18	4.6%<br>18	12	86	21.8%<br>86	69	14	166	42.1%<br>166
9	Revista CUIDARTE	18	106	81	189	80	118	10	10	99	25.1%<br>99	27	30	7.6%<br>30	63	1	0.3%<br>1	2	114	28.9%<br>114	79	4	131	33.2%<br>131

10	Avances en Enfermería	24	138	73	167	67	103	19	19	74	18.8%<br>74	35	26	6.6%<br>26	58	2	0.5%<br>2	11	88	22.3%<br>88	68	15	168	42.6%<br>168
11	Enfermería Clínica	1	17	59	156	54	101	2	2	148	37.6%<br>148	7	77	19.5%<br>77	1	31	7.9%<br>31	5	99	25.1%<br>99	80	2	124	31.5%<br>124
12	Cultura de los Cuidados	16	97	29	123	49	101	14	14	87	22.1%<br>87	23	34	8.6%<br>34	13	13	3.3%<br>13	21	75	19%<br>75	56	26	190	48.2%<br>190
13	Investigación en enfermería: imagen y desarrollo	54	253	60	156	60	102	47	47	38	9.6%<br>38	57	14	3.6%<br>14	61	2	0.5%<br>2	38	52	13.2%<br>52	31	50	232	58.9%<br>232
14	Temperamentvm	62	304	42	135	79	112	60	60	29	7.4%<br>29	60	13	3.3%<br>13	46	5	1.3%<br>5	60	34	8.6%<br>34	5	78	265	67.3%<br>265
15	Gerokomos	27	140	15	107	64	103	23	23	64	16.2%<br>64	24	33	8.4%<br>33	19	10	2.5%<br>10	31	60	15.2%<br>60	40	43	221	56.1%<br>221
16	Revista Ética de los Cuidados	36	181	63	158	61	102	27	27	60	15.2%<br>60	58	13	3.3%<br>13	40	6	1.5%<br>6	24	70	17.8%<br>70	50	32	202	51.3%<br>202
17	Archivos de la Memoria	66	318	49	148	70	104	57	57	30	7.6%<br>30	71	8	2.0%<br>8	47	5	1.3%<br>5	69	30	7.6%<br>30	9	74	256	65%<br>256
18	Revista Tesela	61	293	61	156	72	106	58	58	30	7.6%<br>30	72	8	2.0%<br>8	54	3	0.8<br>3	45	42	10.7%<br>42	19	64	247	62.7%<br>247
19	Revista Cubana de Enfermería	17	98	24	116	42	99	15	15	84	21.3%<br>84	5	84	21.3%<br>84	27	8	2%<br>8	23	71	18%<br>71	55	28	191	48.5%<br>191
20	Revista de Enferme	68	328	39	132	69	104	65	65	23	5.8%<br>23	45	23	5.8%<br>23	64	1	0.3%<br>1	74	24	6.1%<br>24	3	80	269	68.3%<br>269

	ría del Instituto Mexicano del Seguro Social																							
21	Metas de Enfermería	4	43	37	129	39	97	6	6	122	31.0% 122	3	122	31.0% 122	4	21	5.3% 21	14	81	2.6% 81	67	16	170	43.1% 170
22	Enfermería Comunitaria. Revista internacional de cuidados de salud familiar y comunitaria	6	51	46	145	53	101	8	8	117	29.7% 117	4	117	29.7% 117	24	9	2.3% 9	7	93	23.6% 93	75	8	150	38.1% 150
23	Matronas Profesión	50	248	16	108	76	109	61	61	28	7.1% 28	32	28	7.1% 28	48	4	1% 4	44	46	11.7% 46	18	63	247	62.7% 247
24	Revista Rol de Enfermería	2	17	43	139	74	108	3	3	135	34.3% 135	2	135	34.3% 135	2	24	6.1% 24	4	102	25.9% 102	77	6	141	35.8% 141
25	Enfermería Docente	30	152	5	98	63	103	35	35	48	12.2% 48	15	48	12.2% 48	35	6	1.5% 6	28	64	16.5% 64	44	39	213	54.1% 213
26	Biblioteca Lascasas	39	211	10	102	55	102	43	43	41	10.4% 41	19	41	10.4% 41	42	5	1.3% 5	46	41	10.4% 41	21	61	244	61.9% 244
27	Revista da Escola de Enfermagem da USP	13	76	9	102	44	100	17	17	81	20.6% 81	13	52	13.2% 52	8	18	4.6% 18	17	79	20.1% 79	62	21	184	46.7% 184

28	Texto & Contexto: Enfermagem	14	89	26	117	48	101	18	18	78	19.8%<br>78	14	52	13.2%<br>52	25	9	2.3%<br>9	13	82	20.8%<br>82	64	19	178	45.2%<br>178
29	Escola Anna Nery Revista de Enfermagem	28	151	2	82	36	96	34	34	50	12.7%<br>50	1	323	8.1%<br>32	31	7	1.8%<br>7	39	50	12.7%<br>50	36	46	228	57.9%<br>228
30	Revista Latino-Americana de Enfermagem	8	65	21	115	37	97	11	11	93	23.6%<br>93	11	55	14.0%<br>55	11	15	3.8%<br>15	15	80	20.3%<br>80	65	17	174	44.2%<br>174
31	Brasileira de Enfermagem	10	71	6	101	41	99	16	16	82	20.8%<br>82	12	55	14.0%<br>55	7	18	4.6%<br>18	16	79	20.1%<br>79	63	20	179	45.4%<br>179
32	Revista Gaúcha de Enfermagem	33	167	7	101	34	94	32	32	55	14.0%<br>55	18	41	10.4%<br>41	28	8	2%<br>8	41	46	11.7%<br>46	34	48	230	58.4%<br>230
33	ACTA Paulista de Enfermagem	31	156	25	116	32	92	26	26	63	16.0%<br>63	21	40	10.2%<br>40	32	7	1.8%<br>7	37	52	13.5%<br>52	43	40	215	54.6%<br>215
34	Ciência, Cuidado e Saúde	22	129	19	111	58	102	24	24	64	16.2%<br>64	26	31	7.9%<br>31	21	10	2.5%<br>10	25	69	17.5%<br>69	51	33	202	51.3%<br>202
35	Revista Eletrônica de Enfermagem	19	110	17	109	57	102	20	20	71	18.0%<br>71	20	40	10.2%<br>40	18	10	2.5%<br>10	22	72	18.3%<br>72	53	30	198	50.3%<br>198
36	Revista da Rede de Enferma	46	231	4	93	47	101	42	42	42	10.7%<br>42	39	25	6.3%<br>25	12	15	3.8%<br>15	67	30	7.6%<br>30	13	71	250	63.5%<br>250

	gem do<br>Nordeste																							
37	Cogitare<br>Enferma<br>gem	44	227	22	115	38	97	44	44	41	10.4<br>%<br>41	38	26	6.6%<br>26	36	6	1.5%<br>6	51	39	9.9%<br>39	25	58	242	61.4<br>%<br>242
38	Revista<br>de<br>Enferma<br>gem da<br>UFSM	48	237	35	127	43	99	38	38	43	10.9<br>%<br>43	40	24	6.1%<br>24	44	5	1.3%<br>5	53	37	9.34<br>%<br>37	22	62	244	61.9<br>%<br>244
39	Revista<br>Mineira<br>de<br>Enferma<br>gem	53	252	36	128	65	103	39	39	43	10.9<br>%<br>43	47	20	5.1%<br>20	38	6	1.5%<br>6	59	34	8.6%<br>34	15	69	249	63.2<br>%<br>249
40	Revista<br>Enferma<br>gem em<br>Foco	42	215	34	126	50	101	37	37	45	11.4<br>%<br>45	51	18	4.6%<br>18	30	8	2%<br>8	42	46	11.7<br>%<br>46	28	55	237	60.2<br>%<br>237
41	Revista<br>SOBEC<br>C	71	336	72	162	75	108	62	62	26	6.6%<br>26	80	5	1.3%<br>5	56	3	0.8%<br>3	63	33	8.4%<br>33	7	75	257	65.2<br>%<br>257
42	Revista<br>de<br>Enferma<br>gem<br>Referênc<br>ia	59	275	28	120	77	109	56	56	31	7.9%<br>31	50	19	4.8%<br>19	39	6	1.5%<br>6	57	36	9.1%<br>36	10	73	252	64%<br>252
43	Revista<br>de<br>Enferma<br>gem do<br>Centro-<br>Oeste<br>Mineiro	77	360	44	139	52	101	75	75	14	3.6%<br>14	75	8	2.0%<br>8	50	4	1%<br>4	79	20	5.1%<br>20	2	81	275	69.8<br>%<br>275
44	Revista<br>de<br>Enferma<br>gem<br>UFPE<br>On Line	45	230	1	70	62	103	49	49	37	9.4%<br>37	30	29	7.4%<br>29	3	24	6.1%<br>24	71	26	6.6%<br>26	6	77	260	66%<br>260
45	Revista<br>Baiana<br>de	57	267	13	105	56	102	50	50	36	9.1%<br>36	52	18	4.6%<br>18	17	11	2.8%<br>11	72	26	6.6%<br>26	8	76	257	65.2<br>%<br>257

	Enfermagem																							
46	Revista de Enfermagem da Universidade Federal do Piauí	74	347	55	152	71	105	66	66	23	5.8% 23	67	10	2.5% 10	62	2	0.5% 2	73	26	6.6% 26	4	79	268	68% 268
47	Revista de Pesquisa: Cuidado é Fundamental	49	242	8	101	68	104	53	53	35	8.9% 35	43	24	6.1% 24	23	10	2.5% 10	55	36	9.1% 36	14	68	249	63.2% 249
48	Revista de Enfermagem e Atenção a Saùde	63	310	69	161	66	103	59	59	30	7.6% 30	63	12	3.0% 12	68	1	0.3% 1	54	37	9.4% 37	16	66	248	62.9% 248
49	International Journal of Nursing Studies	9	66	80	188	81	127	4	4	128	32.5% 128	9	64	16.2% 64	51	3	0.8% 3	1	126	32% 126	82	1	90	22.8% 90
50	Journal of Nursing Scholarship	25	138	32	125	29	86	28	28	60	15.2% 60	22	38	9.6% 38	43	5	1.3% 5	27	68	17.3% 68	66	18	174	44.2% 174
51	European Journal of Cardiovascular Nursing	32	160	50	149	31	89	33	33	53	13.5% 53	37	26	6.6% 26	59	2	0.5% 2	20	78	19.8% 78	71	11	161	40.9% 161

52	Nursing Outlook	40	212	51	151	17	81	36	36	46	11.7%<br>46	53	17	4.3%<br>17	52	3	0.8%<br>3	40	50	12.7%<br>50	52	31	201	51%<br>201
53	European Journal of Cancer Care	41	214	53	152	21	82	46	46	39	9.9%<br>39	48	19	4.8%<br>19	65	1	0.3%<br>1	32	59	15%<br>59	60	23	187	47.5%<br>187
54	Birth-issues in perinatal care	78	361	68	160	8	77	79	79	12	3.0%<br>12	76	8	2.0%<br>8	72	1	0.3%<br>1	78	21	5.3%<br>21	27	56	238	60.4%<br>238
55	Journal of advanced Nursing	5	48	23	116	59	102	13	13	92	23.4%<br>92	10	57	14.5%<br>57	14	11	2.8%<br>11	6	97	24.6%<br>97	78	5	135	34.3%<br>135
56	Worldviews on Evidence-Based Nursing	56	261	40	134	16	81	63	63	26	6.6%<br>26	55	17	4.3%<br>17	53	3	0.8%<br>3	49	40	10.2%<br>40	41	41	216	54.8%<br>216
57	Journal of Cardiovascular Nursing	37	197	47	146	27	85	40	40	43	10.9%<br>43	29	29	7.4%<br>29	74	0	0%<br>0	30	61	15.5%<br>61	58	24	188	47.7%<br>188
58	Nurse Education Today	23	137	11	103	11	79	30	30	58	14.7%<br>58	25	32	8.1%<br>32	20	10	2.5%<br>10	35	52	13.2%<br>52	57	27	190	48.2%<br>190
59	American Journal of Critical Care	21	119	27	120	14	81	25	25	64	16.2%<br>64	34	27	6.9%<br>27	22	10	2.5%<br>10	26	69	17.5%<br>69	72	12	161	40.9%<br>161
60	International Journal of Mental Health Nursing	20	117	12	105	2	75	29	29	59	15.0%<br>59	36	26	6.6%<br>26	10	16	4.1%<br>16	29	62	15.7%<br>62	70	13	163	41.4%<br>163

61	Journal of Family Nursing	35	180	18	110	13	81	45	45	40	10.2%<br>40	41	24	6.1%<br>24	29	8	2%<br>8	36	52	13.2%<br>52	54	29	194	49.2%<br>194
62	Australian Critical Care	60	279	14	105	12	81	72	72	17	4.3%<br>17	62	13	3.3%<br>13	26	9	2.3%<br>9	68	30	7.6%<br>30	32	51	232	58.9%<br>232
63	Journal of Tissue Viability	79	361	45	143	20	82	80	80	11	2.8%<br>11	77	8	2.0%<br>8	55	3	0.8%<br>3	82	15	3.8%<br>15	17	67	248	62.9%<br>248
64	Journal of Nursing Management	34	174	3	90	9	78	41	41	43	10.9%<br>43	31	28	7.1%<br>28	16	11	2.8%<br>11	48	40	10.2%<br>40	45	38	212	53.8%<br>212
65	Nursing Ethics	43	225	33	125	15	81	55	55	33	8.4%<br>33	59	13	3.3%<br>13	33	7	1.8%<br>7	43	46	11.7%<br>46	48	35	208	52.8%<br>208
66	Cancer Nursing	58	273	30	124	3	75	67	67	22	5.6%<br>22	68	10	2.5%<br>10	34	7	1.8%<br>7	62	33	8.4%<br>33	42	42	216	54.8%<br>216
67	Journal of Human Lactation	76	353	65	159	78	110	82	82	8	2.0%<br>8	78	7	1.8%<br>7	73	1	0.3%<br>1	50	40	10.2%<br>40	12	70	250	63.5%<br>250
68	Women and Birth	73	345	52	151	5	75	73	73	16	4.1%<br>16	66	11	2.8%<br>11	69	1	0.3%<br>1	80	18	4.6%<br>18	26	57	241	61.2%<br>241
69	World Psychiatry	75	347	76	171	7	76	69	69	20	5.1%<br>20	73	8	2.0%<br>8	78	0	0%<br>0	75	24	6.1%<br>24	33	52	232	58.9%<br>232
70	Diabetes Care	26	138	66	160	30	88	21	21	70	17.8%<br>70	33	27	6.9%<br>27	57	2	0.5%<br>2	18	79	20.1%<br>79	74	9	157	39.8%<br>157
71	Stroke	72	336	74	168	10	78	74	74	15	3.8%<br>15	74	8	2.0%<br>8	79	0	0%<br>0	64	33	8.4%<br>33	38	45	224	56.9%<br>224
72	American Journal of Clinical	47	231	62	157	18	81	51	51	36	9.1%<br>36	46	22	5.6%<br>22	75	0	0%<br>0	34	56	14.2%<br>56	59	25	188	47.7%<br>188

	Nutrition																							
73	International Journal of Obesity	51	249	48	148	4	75	54	54	34	8.6% 34	54	17	4.3% 17	60	2	0.5% 2	47	41	10.4% 41	49	34	207	52.5% 207
74	Resucitation	69	328	57	154	6	75	77	77	13	8.4% 13	70	10	2.5% 10	71	1	0.3% 1	66	31	7.9% 31	39	44	222	56.3% 222
75	International Journal of Behavioral Nutrition and Physical Activity	70	328	64	159	28	85	70	70	20	5.1% 20	69	10	2.5% 10	70	1	0.3% 1	65	31	7.9% 31	29	54	235	59.6% 235
76	Nutrition Reviews	67	320	67	160	26	84	71	71	18	4.6% 18	65	11	2.8% 11	77	0	0% 0	58	35	8.9% 35	35	49	230	58.4% 230
77	Current Opinion in HIV and AIDS	82	386	75	170	25	83	81	81	11	2.8% 11	79	6	1.5% 6	80	0	0% 0	81	18	4.6% 18	20	65	247	62.7% 247
78	Advances in Nutrition	65	316	70	161	19	81	68	68	21	5.3% 21	64	11	2.8% 11	76	0	0% 0	61	34	8.6% 34	37	47	228	57.9% 228
79	Journal of the American Medical Directors Association	81	377	77	175	22	82	78	78	13	3.3% 13	81	5	1.3% 5	81	0	0% 0	77	22	5.6% 22	24	60	243	61.7% 243
80	Journal of Pain and	64	315	54	152	24	83	64	64	24	6.1% 24	61	13	3.3% 13	67	1	0.3% 1	70	30	7.6% 30	30	53	234	59.4% 234

	Symptom Management																						
81	Journal of Palliative Medicine	52	251	56	153	1	74	48	48	38	9.6% 38	49	19	4.8% 19	66	1	0.3% 1	52	38	9.6% 38	47	36	209	53% 209
82	Patient	80	375	78	178	23	82	76	76	14	3.6% 14	82	4	1.0% 4	82	0	0% 0	76	23	5.8% 23	23	59	243	61.7% 243
